# Cerium oxide nanoparticles inhibit differentiation of neural stem cells

**DOI:** 10.1038/s41598-017-09430-8

**Published:** 2017-08-24

**Authors:** Anda R. Gliga, Karin Edoff, Fanny Caputo, Thomas Källman, Hans Blom, Hanna L. Karlsson, Lina Ghibelli, Enrico Traversa, Sandra Ceccatelli, Bengt Fadeel

**Affiliations:** 1 Division of Molecular Toxicology, Karolinska Institutet, Stockholm, Sweden; 2 Division of Biochemical Toxicology, Institute of Environmental Medicine, Karolinska Institutet, Stockholm, Sweden; 3 Department of Neuroscience, Karolinska Institutet, Stockholm, Sweden; 4 Department of Biology, University of Rome ‘Tor Vergata’, Rome, Italy; 5 Department of Chemical Science and Technology, University of Rome ‘Tor Vergata’, Rome, Italy; 6 Department of Medical Biochemistry and Microbiology, Uppsala University, Uppsala, Sweden; 7 Bioinformatics Infrastructure for Life Sciences, Uppsala University, Uppsala, Sweden; 8 Science for Life Laboratory, Royal Institute of Technology, Solna, Sweden; 9 International Research Center for Renewable Energy, Xi’an Jiaotong University, Xi’an, China

## Abstract

Cerium oxide nanoparticles (nanoceria) display antioxidant properties and have shown cytoprotective effects both *in vitro* and *in vivo*. Here, we explored the effects of nanoceria on neural progenitor cells using the C17.2 murine cell line as a model. First, we assessed the effects of nanoceria *versus* samarium (Sm) doped nanoceria on cell viability in the presence of the prooxidant, DMNQ. Both particles were taken up by cells and nanoceria, but not Sm-doped nanoceria, elicited a temporary cytoprotective effect upon exposure to DMNQ. Next, we employed RNA sequencing to explore the transcriptional responses induced by nanoceria or Sm-doped nanoceria during neuronal differentiation. Detailed computational analyses showed that nanoceria altered pathways and networks relevant for neuronal development, leading us to hypothesize that nanoceria inhibits neuronal differentiation, and that nanoceria and Sm-doped nanoceria both interfere with cytoskeletal organization. We confirmed that nanoceria reduced neuron specific β3-tubulin expression, a marker of neuronal differentiation, and GFAP, a neuroglial marker. Furthermore, using super-resolution microscopy approaches, we could show that both particles interfered with cytoskeletal organization and altered the structure of neural growth cones. Taken together, these results reveal that nanoceria may impact on neuronal differentiation, suggesting that nanoceria could pose a developmental neurotoxicity hazard.

## Introduction

Cerium oxide nanoparticles (nanoceria) are inorganic, rare earth nanoparticles possessing catalytic antioxidant activity. The application of nanoceria in various industrial settings is well-established^1^. However, their potential use as *bona fide* antioxidants in biological systems has emerged more recently^2, 3^. In particular, nanoceria has shown promise in animal models of retinal degeneration^4, 5^ and other recent studies have indicated that nanoceria treatment decreases infarct volume in a rat model of ischemic stroke^6^ and reduces motor symptoms in a mouse model of multiple sclerosis, a debilitating autoimmune degenerative disease of the central nervous system^7^. Several *in vitro* studies have also indicated that nanoceria protects primary cortical neurons and primary spinal cord neurons against oxidative stress when cultured *ex vivo* ^8–10^. Moreover, cardioprotective effects of cerium oxide nanoparticles were described in a murine model of cardiomyopathy^11^ and nanoceria was previously shown to protect murine cardiac progenitor cells from oxidative stress up to 7 days after an initial 24 h exposure^12^.

Despite the large body of literature showing beneficial effects of nanoceria^13, 14^, concerns have been raised over the potential toxicity following nanoceria exposure, including inadvertent human exposure resulting from the fact that nanoceria is used as a diesel fuel additive^15^. Indeed, as shown in a recent inhalation study in rats, diesel emissions generated with cerium oxide additives induce more adverse pulmonary effects on a mass basis than diesel emissions alone^16^. In addition, Hardas *et al*.^17^ reported that a single intravenous administration of nanoceria (5 nm) induced pro-oxidant effects in the brain at 30-days post-exposure without crossing the blood-brain barrier. In another study, the same group revealed that a single intravenous administration of nanoceria (30 nm) elicited a so-called hierarchical oxidative stress response in the rat hippocampus with a peak at day 30 and resolution at day 90 post-exposure^18^. In addition, *in vitro* studies have shown that nanoceria induces apoptosis and autophagy in primary human monocytes in a manner that is not dependent on ROS production^19^, which is in apparent contradiction to the previous observation that nanoceria prevents oxidative stress-dependent apoptosis in human monocyte/lymphocyte cell lines^20^. Induction of autophagy by nanoceria has also been documented by others, albeit in the absence of apoptosis or cytotoxicity^21^.

The contradictory observations in the literature with regards to effects of nanoceria could be explained by the application of different doses, using different model systems, but could also be due to the different intrinsic properties of the particles such as size and shape^22^ as well as surface chemistry, which may determine the intrinsic antioxidant properties^23^. Indeed, in a recent study, the authors found that the pulmonary inflammation and fibrosis in rats was reduced when the nanoceria was coated with a thin layer of amorphous silica^24^. Moreover, as highlighted in a study using an environmentally relevant organism (alga), the percentage of surface content of Ce^3+^ is an important determinant of toxicity of nanoceria^25^. The effect of surface valence states at nanoceria coated surfaces on cell proliferation has been previously noted^26^.

Oxidative stress has been associated with several neurodegenerative diseases, but it is still unclear whether it is the initiating event or a secondary event involved in disease propagation^27^. Nonetheless, antioxidant therapies are under consideration for neurodegenerative diseases, with the aim either to chelate already formed reactive oxygen species (ROS) or prevent their generation^28^. On the other hand, controlled generation of ROS is involved in cellular signaling^29^ and has an important role in maintaining genomic stability in stem cells^30^ as well as in neuronal development and differentiation^31^. Consequently, a reduction in intracellular ROS levels could severely impair neurogenesis^32^. This raises the question as to whether an antioxidant could impact negatively on differentiation of neural stem cells, despite having beneficial effects on neuronal survival.

Here, we investigated the effects of nanoceria on neuronal survival in the face of an oxidative challenge as well the putative effects on neuronal differentiation. To this end, we used the multipotent murine C17.2 neural stem cell line which is considered a good model for neurotoxicity studies as these cells can generate a mixed culture of neurons and glial cells upon differentiation^33^. Neural stem cells are present during neuronal development but are also found in adult brains in stem cells niches, making this model relevant both from a developmental toxicology perspective and for neurotoxicity targeting the adult brain^34^. First, we investigated if the reported antioxidant protective effects are valid for neural stem cells. Next, we evaluated the effects of nanoceria during neuronal differentiation using a next-generation sequencing approach to explore the gene expression changes at early (day 1) and late (day 7) differentiation time-points. In order to distinguish potential antioxidant effects we used a traditional antioxidant, N-acetylcysteine (NAC) as a control, along with nanoceria doped with another rare earth element, samarium (Sm) as a particle control. Our previous studies have shown that Sm-doped nanoceria displays a blunted antioxidant effect^20^. Fluorescence microscopy and enzyme-linked immunosorbent assay based analysis of markers of neural and neuroglial differentiation along with super-resolution microscopy (SIM and STED) was performed to validate the RNA-Seq results. Our studies show that nanoceria inhibits neural stem cell differentiation.

## Results and Discussion

### CeO_2_ and Sm-CeO_2_ are non-cytotoxic for proliferating neural stem cells

In the present study, we evaluated the effects of nanoceria (CeO_2_) and Sm-doped nanoceria (Sm-CeO_2_, particle control) synthesized as previously described^20, 35^. Doping is a relatively easy way to change the surface chemistry of nanoceria^36^. Our previously published procedure of doping allows for the alteration of the Ce^3+^/Ce^4+^ ratio and the associated Ce^3+^/Ce^4+^ redox catalytic activities without affecting oxygen vacancies (Supplementary Figure 1). Previous studies showed that the 20% Sm-doped nanoceria display a reduced antioxidant capacity, both under acellular conditions and when tested in cells (*i.e*., human leukocyte cell lines and keratinocytes)^20, 37^. As reported in the latter study, doping of the particles with Sm results in a slight increase in primary particle size, but it was concluded that this does not influence the results as normalization per surface area of the doped nanoparticles did not substantially affect the results with respect to antioxidant effects^20^. The same study indicated that CeO_2_ doped with 20% Sm bear half of the antioxidant activity in acellular conditions and confers 9 times less antioxidant protection in cells as compared to CeO_2_ alone. The levels of Ce^3+^ were evaluated in our previous work and were found to be 21% for CeO_2_ and 6% for the Sm-CeO_2_. Altogether, the evidence indicated that the antioxidant effects of nanoceria correlated with the fraction of Ce^3+^ and not with oxygen vacancies^20^. In the present study, we determined particle size and zeta potential in H_2_O, in C17.2 culture medium, and in cell differentiation medium. CeO_2_ nanoparticles exhibited a higher degree of agglomeration in the C17.2 medium when compared to the Sm-doped CeO_2_ nanoparticles, while in the serum-free differentiation medium the size of the agglomerates of both particles was similar. Notably, the particles displayed negative zeta potential values in all test media and the surface charge did not change upon doping of the particles with Sm (Supplementary Figure 1).

**Figure 1:**
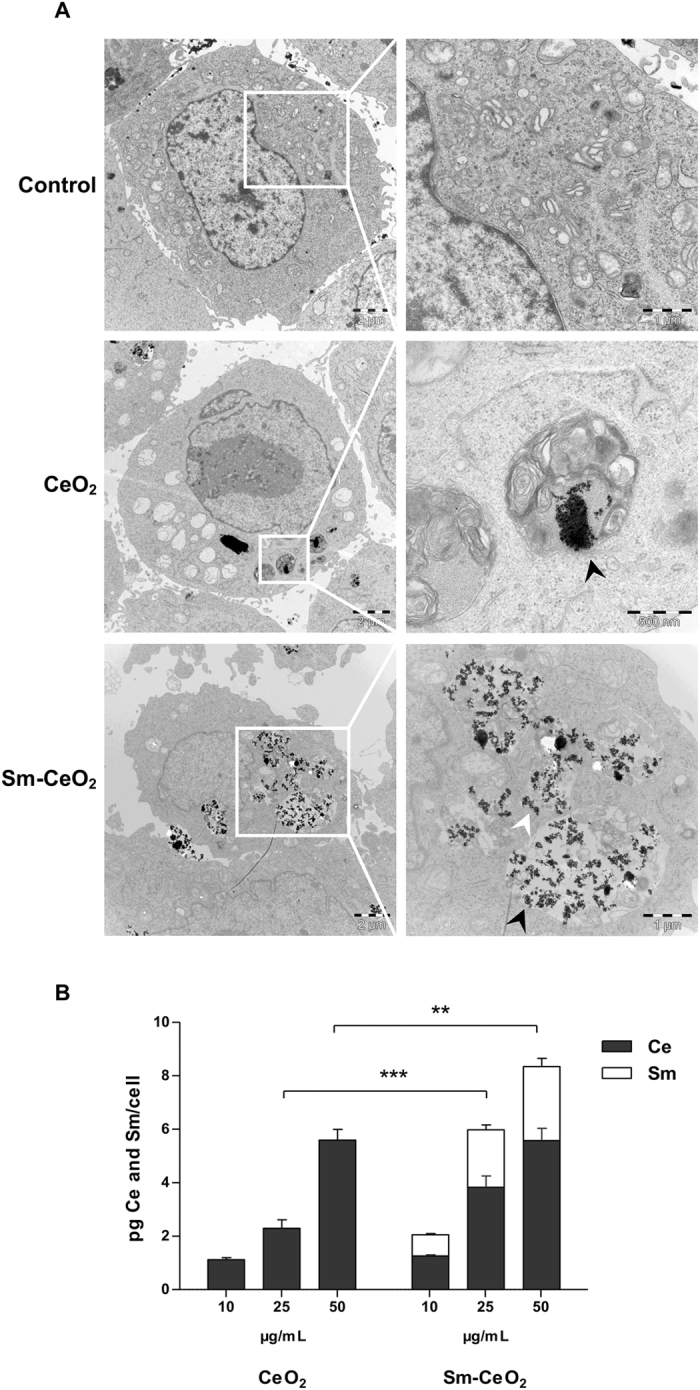
Cellular uptake of CeO_2_ and Sm-CeO_2_ nanoparticles in C17.2 cells. (**A**) C17.2 cells were exposed to 50 μg/mL CeO_2_ or Sm-CeO_2_ nanoparticles for 24 h. Intracellular localization was investigated by TEM. Both nanoparticles were taken up and localized in membrane-bound vesicles (black arrowheads) or free in the cytoplasm (white arrowhead). (**B**) C17.2 cells were exposed to 10, 25 and 50 μg/mL CeO_2_ and Sm-CeO_2_ nanoparticles for 24 h. The cellular Ce and Sm content was quantified by ICP-MS and the cellular dose was expressed as pg Ce and Sm per cell. For Ce the quantification was based on the ^140^Ce and ^142^Ce isotopes while for Sm the quantification was based on the ^147^Sm, ^149^Sm, ^152^Sm, ^154^Sm isotopes. Results are presented as mean values ± S.D. (n = 3). Significant results for the comparison of the total cellular metal content (sum of Ce and Sm) are marked with asterisks (**p-value < 0.01, ***p-value < 0.001).

Next, in order to test the cytotoxicity of the CeO_2_ and Sm-CeO_2_ nanoparticles, proliferating C17.2 cells were exposed to a dose range of 5–100 µg/mL of nanoparticles for 48 h and a cell viability test was performed. The results showed that both nanoparticles were non-cytotoxic (Supplementary Figure 2). This observation is in accordance with numerous other studies in the literature documenting a lack of cytotoxicity for nanoceria in various other cell lines^38–40^. We then proceeded to investigate the intracellular localization (by transmission electron microscopy, TEM) and cellular uptake of the nanoparticles at 24 h of exposure (by inductively coupled plasma mass spectrometry, ICP-MS). TEM imaging (Fig. 1A) showed that both particles were taken up by the proliferating C17.2 cells and that the particles were predominantly localized in membrane bound structures and to a lesser extent were found free in the cytoplasm. There was no indication of any nanoparticles in the cell nucleus. Quantification by ICP-MS indicated that both nanoparticles were taken up in a dose-dependent manner (Fig. 1B). Additionally, when comparing the total cellular metal content (*i.e*., the sum of Ce and Sm), the Sm-CeO_2_ nanoparticles appeared to be taken up to a higher extent at the higher concentrations tested when compared to the CeO_2_ nanoparticles. However, the amount of internalized Ce was comparable for the parental and doped nanoparticles. Overall, these results show that both CeO_2_ and the Sm-doped CeO_2_ nanoparticles are readily internalized by murine neural progenitor cells and that this was not accompanied by cell death.

### CeO_2_, but not Sm-CeO_2_ provides a transient protection against the prooxidant, DMNQ

**Figure 2:**
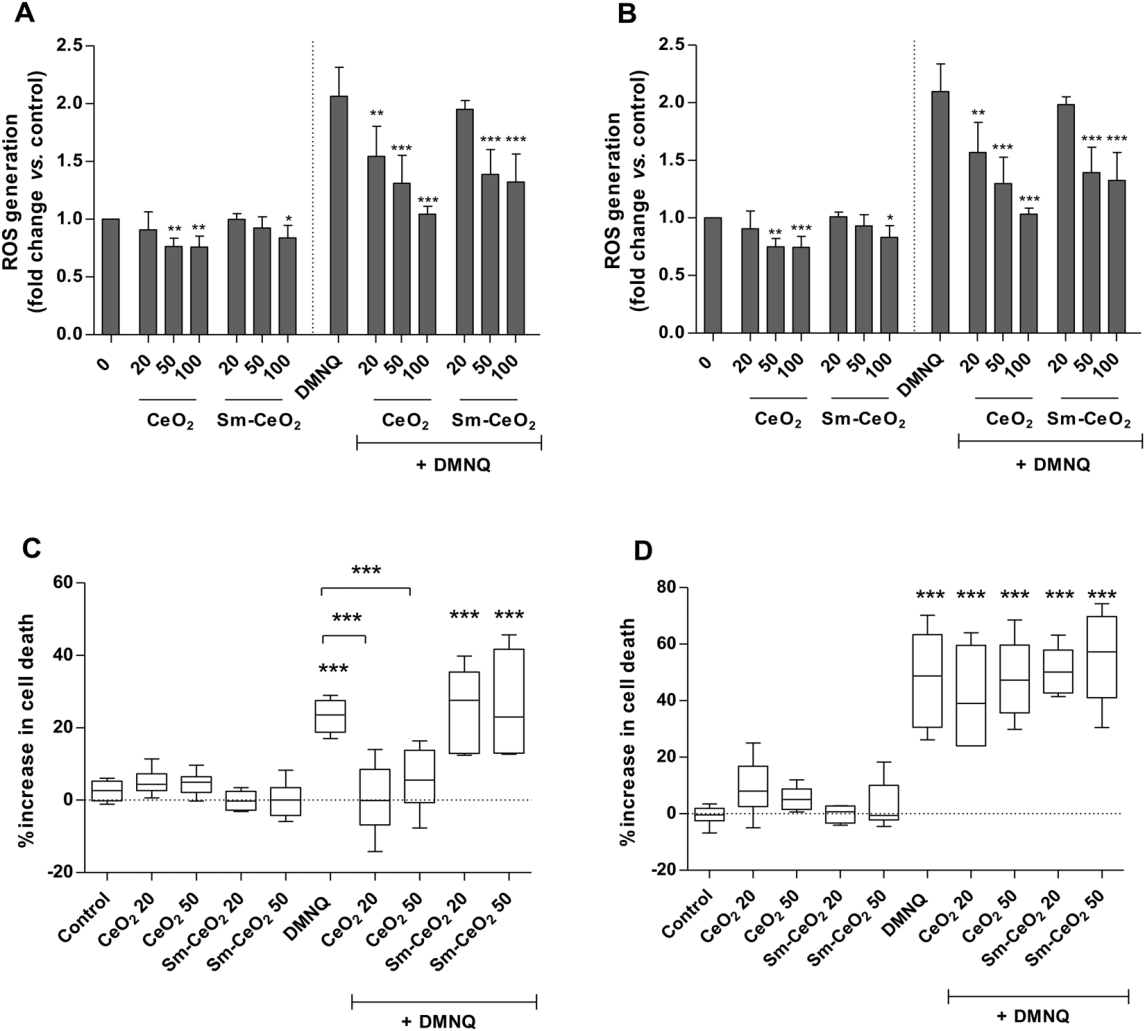
CeO_2_ reduce ROS generation and delay ROS-induced cell death in C17.2 cells. (**A**,**B**) ROS generation after exposure to the oxidative stress inducer, DMNQ in the presence of CeO_2_ or Sm-CeO_2_ nanoparticles was investigated using the DCFH-DA assay. Cells were incubated with CeO_2_ or Sm-CeO_2_ (20, 50, 100 µg/mL) for 4 h, loaded with DCFH-DA and challenged with DMNQ (10 μM). ROS formation was assessed on a plate reader (Ex 485 nm/Em 535 nm) at 8 (**A**) and 12 h (**B**). ROS induction was expressed as fold change *versus* the control. Results are presented as mean values ± S.D. (n = 5). Significant results are indicated with asterisks (*p-value <0.05, **p-value <0.01, ***p-value <0.001). (**B**,**C**) C17.2 cells were exposed to CeO_2_ and Sm-CeO_2_ nanoparticles (20, 50 μg/mL) for 4 h, then challenged with DMNQ (10 μM). Light microscopy images were taken every 30 min over 12 h. The images taken at 0 h, 8 h and 12 h were scored in terms of number of flat cells, mitotic cells and dead cells. Results are expressed as % increased cell death after 8 h (**B**) and 12 h (**C**) compared to time-point 0 h. Results are presented as box plots of 6 replicates (the box corresponds to the interquartile range, the line inside the box is plotted at the median and the whiskers show the minimum and maximum values). Significant results are marked with asterisks (***p-value <0.001).

CeO_2_ nanoparticles have previously been reported to reduce basal intracellular ROS levels and to diminish etoposide-induced ROS formation in human cell lines and these protective effects were absent for the Sm-CeO_2_ nanoparticles^18^. In addition, nanoceria was also found to quench ROS and inhibit production of inflammatory mediators in the J774A.1 macrophage cell line^41^. Here, we aimed to evaluate whether similar antioxidant effects could be observed in the C17.2 neural progenitor cell line. To this end, we exposed C17.2 cells to CeO_2_ or Sm-doped CeO_2_ nanoparticles for 4 h, then loaded the cells with the DCFH-DA probe, followed by exposure to 2,3-dimethoxy-1,4-naphthoquinone (DMNQ, 10 µM). DMNQ is a known inducer of oxidative stress and has been shown previously to trigger cell death in the C17.2 cell line^42^. ROS generation was evaluated at 2, 4, 6, 8 and 12 h. The results showed that CeO_2_ nanoparticles significantly and dose-dependently reduced DMNQ-induced ROS generation at all evaluated time-points, starting at 20 µg/mL (Fig. 2A,B and Supplementary Figure 3). Additionally, at the highest concentrations (50 and 100 µg/mL) basal ROS levels were also considerably reduced. However, when the Sm-CeO_2_ nanoparticles were evaluated protection against DMNQ-induced ROS generation was noted only for the highest concentrations (Fig. 2A,B and Supplementary Figure 3). These results thus suggested that CeO_2_ nanoparticles have an antioxidant effect in the C17.2 neural progenitor cell line. The Sm-CeO_2_ nanoparticles also display an antioxidant effect, but only at high concentrations (50 or 100 µg/mL). Next, we asked whether the observed protection against DMNQ-induced ROS generation would translate into a reduction in cell death. We treated C17.2 cells with CeO_2_ nanoparticles (20 and 50 µg/mL) and Sm-CeO_2_ nanoparticles (20 and 50 µg/mL) for 4 h and then challenged cells with DMNQ (10 µM). Cell viability was determined by microscopic assessment of cell numbers and cell morphology (Cell-IQ assay). We noted that there was no significant increase in cell death in DMNQ-treated cells up to 6 h of exposure (data not shown) and therefore proceeded to score cell viability at later time points. As shown in Fig. 2C, we observed a significant reduction in cell death at 8 h for the CeO_2_ + DMNQ treated samples as compared to the DMNQ alone. The Sm-CeO_2_ did not protect against cell death, indicating that this effect was probably due to the more potent antioxidant properties of nanoceria. Furthermore, at 12 h there was no longer any protection from DMNQ-induced cell death (Fig. 2D). The nanoparticles themselves did not elicit any cell death at any of the time-points tested. We previously reported^42^ that DMNQ triggers mitochondria-dependent apoptosis in C17.2 cells, with characteristic features of apoptosis including the activation of downstream caspases and DNA fragmentation. Thus, while DMNQ is an inducer of oxidative stress, it stands to reason that once the apoptotic program is initiated and reaches the point-of-no-return with activation of the proteolytic caspase cascade^43^, the impact of anti-oxidants would not be as effective. This may explain why the CeO_2_ nanoparticles (and to a lesser extent, the Sm-CeO_2_ nanoparticles) reduced ROS generation at early and late time-points in this model, but prevented cell death induced by DMNQ only at the early time-points. Overall, we conclude that CeO_2_ nanoparticles, but not Sm-doped CeO_2_, delayed ROS-induced cell death, but cytoprotection was only temporary and, ultimately, cell death was not prevented.

### CeO_2_ and Sm-CeO_2_ are non-cytotoxic for differentiating neural stem cells

**Figure 3:**
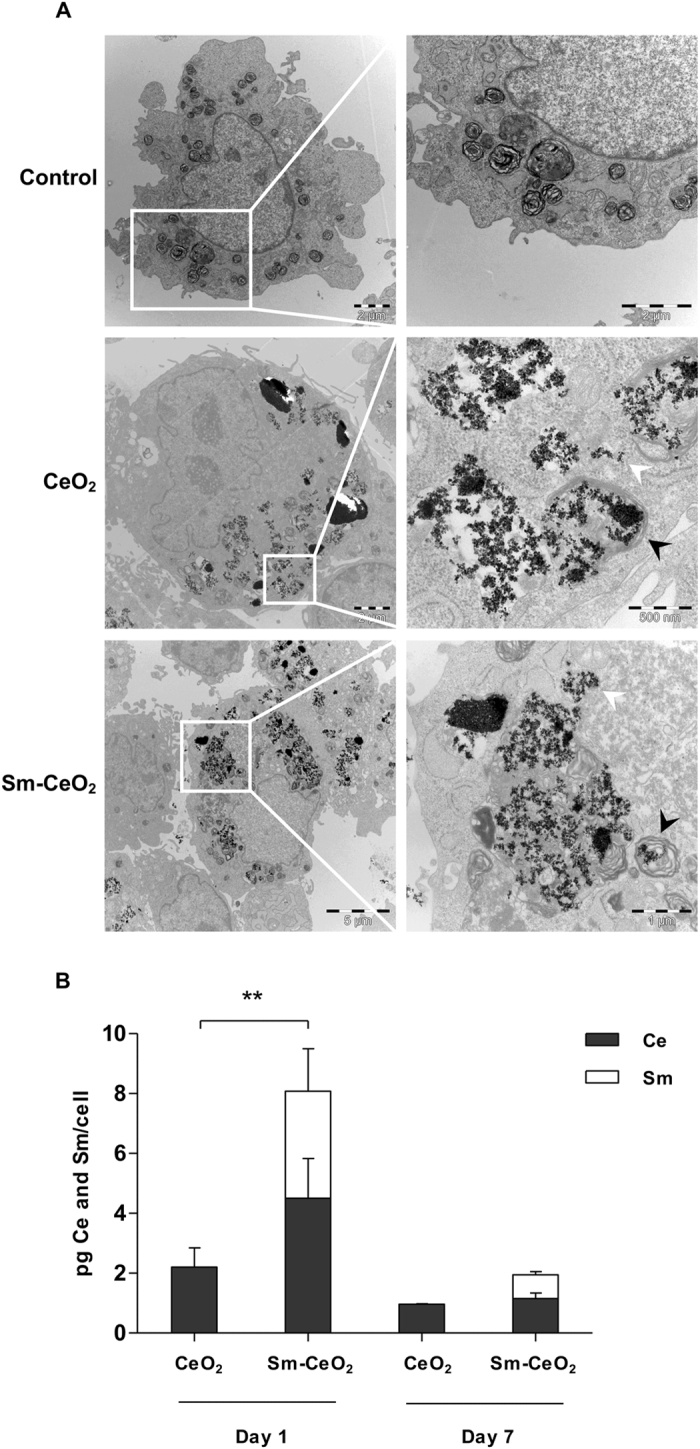
Cellular uptake of CeO_2_ and Sm-CeO_2_ in differentiated C17.2 cells. (**A**) C17.2 cells were differentiated for 7 days in the presence of 50 μg/mL CeO_2_ or Sm-CeO_2_ nanoparticles. TEM imaging demonstrated that both nanoparticles were taken up and localized in membrane-bound vesicles (black arrow heads) or free in the cytoplasm (white arrowheads). (**B**) C17.2 cells were differentiated for 1 and 7 days in the presence of 25 μg/mL CeO_2_ or Sm-CeO_2_ nanoparticles. The cellular Ce and Sm content was quantified by ICP-MS and the cellular dose was expressed as pg Ce and Sm per cell. For Ce the quantification was based on the ^140^Ce and ^142^Ce isotopes while for Sm the quantification was based on the ^147^Sm, ^149^Sm, ^152^Sm, ^154^Sm isotopes. Results are presented as mean values ± S.D. (n = 3). Significant results for the comparison of the total cellular metal content (sum of Ce and Sm) are marked with asterisks (**p-value < 0.01).

Considering the importance of ROS signaling for neuronal differentiation^44^, we proceeded to investigate the effects of the CeO_2_ nanoparticles during neuronal differentiation of the C17.2 neural stem cell line. First, we assessed the impact of CeO_2_ *versus* Sm-CeO_2_ nanoparticles on cell viability/cell proliferation of the C17.2 cells after 7 days of differentiation and found that the particles did not impact negatively on the cells (Supplementary Figure 4A). However, NAC (1 mM) reduced the metabolic activity of the differentiated C17.2 cells, possibly due to an inhibition of cell proliferation. Next, we confirmed that neither treatment induced caspase-3/-7 activation, a feature of apoptosis, in C17.2 cells differentiated for 7 days (Supplementary Figure 4B). The chemotherapeutic agent, etoposide (100 μM) was used as a positive control. We also quantified particle uptake in C17.2 cells after 1 day and 7 days of differentiation and investigated the intracellular localization of the particles. As shown previously for the proliferating C17.2 cells, both CeO_2_ and Sm-CeO_2_ nanoparticles were located in membrane bound vesicles or free in the cytoplasm (Fig. 3A). Moreover, cellular uptake at day 1 was significantly higher for the Sm-CeO_2_ than for the CeO_2_, but at day 7 the uptake of the two particles was comparable (Fig. 3B). The reason for the higher uptake of the Sm-doped nanoparticles is not known, but this implies that the small antioxidant effect that is retained by these particles^20^ is amplified relative to the CeO_2_ nanoparticles (at day 1). There was, overall, a reduction of the cellular dose of particles from day 1 to day 7 which could be due to the previously reported particle dilution effect (*i.e*., nanoparticles internalized by cells are split between daughter cells when the cells divide)^45^.

### RNA-Seq identifies gene expression changes triggered by nanoceria

**Figure 4:**
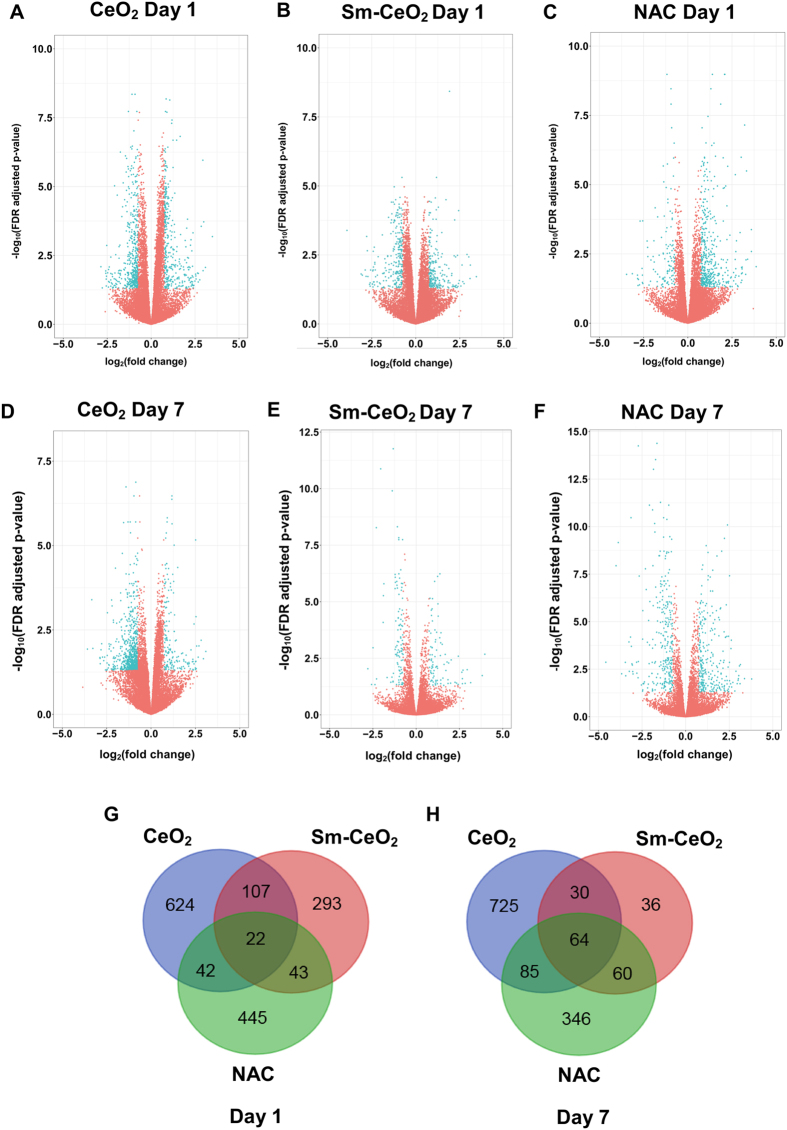
RNA-Seq analysis of differentiating C17.2 neural progenitor cells. (**A**–**F**) Volcano plots illustrating –log_10_(FDR adjusted p-value) in relation to the log_2_(fold change) for the treatments versus control at Day 1 (**A**–**C**) and Day 7 (**D**,**F**). Genes that passed the significance threshold FDR adjusted p-value < 0.05 and the expression cut-off log_2_(fold change) > 0.75 are colored red, while genes outside this range are colored blue. (**G**,**H**) Venn diagrams presenting the number of genes which are differentially expressed (p-value < 0.05 after the FDR multiple testing correction and log_2_(fold change) > 0.75) at day 1 (**G**) and at day 7 (**H**). Cells were exposed to 25 µg/mL CeO_2_ or Sm-CeO_2_, or NAC (1 mM) as described in Methods.

In order to gain further insight into the potential impact of nanoceria on neuronal differentiation, we employed an RNA-Seq approach. RNA-Seq is a relatively recent tool that yields genome-wide quantitative measurements of RNA transcripts^46^. RNA-Seq is technically superior to traditional hybridization techniques such as microarrays as it provides a higher resolution, lower background, offering a far more precise measurement of levels of transcripts and their isoforms, and can be used without preexisting knowledge of the genomic sequence^47^. Additionally, taking a genome-wide approach enables complex pathway and network analyses that may ultimately provide a holistic understanding of how biological systems are affected by nanoparticles or other exposures^47^. Moreover, fold changes generated by RNA-Seq have been shown to be highly correlated with qRT-PCR results^48^ making validation at the gene expression level redundant. RNA-Seq has previously been applied in nanotoxicology to decipher, for instance, the impact of nanoparticle exposure in the eukaryotic green alga, *Chlamydomonas reinhardtii* ^49^, and the effects of low doses of dendrimers on human bronchial epithelial cells^50^. For the present study, C17.2 cells were differentiated for 1 or 7 days in the presence of CeO_2_ nanoparticles (25 μg/mL), Sm-CeO_2_ (25 μg/mL) nanoparticles, or NAC (1 mM). NAC was used as an antioxidant control and was previously reported to interfere with neuronal differentiation in PC12 cells^51^. Three replicates were included for each sample. RNA-Seq was performed by using the Hi-Seq. 2500 Illumina platform and the generated reads were mapped to the annotated mouse genome (version GRCm38). Volcano plots showing the gene expression changes at day 1 (A–C) and day 7 (D–F) for cells incubated with CeO_2_, Sm-CeO_2_, or NAC are shown in Fig. 4. Venn diagrams were plotted for the differentially expressed genes (DEGs) at day 1 and day 7, to illustrate the degree of overlap between the different samples, with a cut-off for FDR adjusted p-value of 0.05 and a cut-off for log_2_(fold change) of 0.75 (Fig. 4G,H). At day 1, the total number of DEGs was 795 (343 upregulated, 452 downregulated) for CeO_2_ nanoparticles, 552 (117 downregulated, 435 upregulated) for NAC and 465 (241 downregulated, 224 upregulated) for the Sm-CeO_2_ nanoparticles (Fig. 4G). At day 7, the total number of DEGs was 904 (740 downregulated, 164 upregulated) for CeO_2_, 555 (280 downregulated, 274 upregulated) for NAC and 190 (103 downregulated, 87 upregulated) for Sm-CeO_2_ (Fig. 4H). The alterations at the gene expression level thus increased with time of culture for the CeO_2_-treated cells, were relatively stable for NAC, and decreased with time for cells exposed to Sm-CeO_2_ nanoparticles. The overlap between the genes with significantly altered expression for cells exposed to CeO_2_ *versus* NAC was low (8% of the genes affected by CeO_2_ at day 1 were shared with NAC, and twice as many at day 7) (Fig. 4G,H). However, this does not exclude the possibility that similar pathways or functional categories of genes were affected by the two treatments and for this reason, and in order to probe the underlying mechanisms, we proceeded with detailed pathway analyses, as described in the following sections.

### RNA-Seq predicts that CeO_2_ negatively affects neuronal differentiation

**Figure 5:**
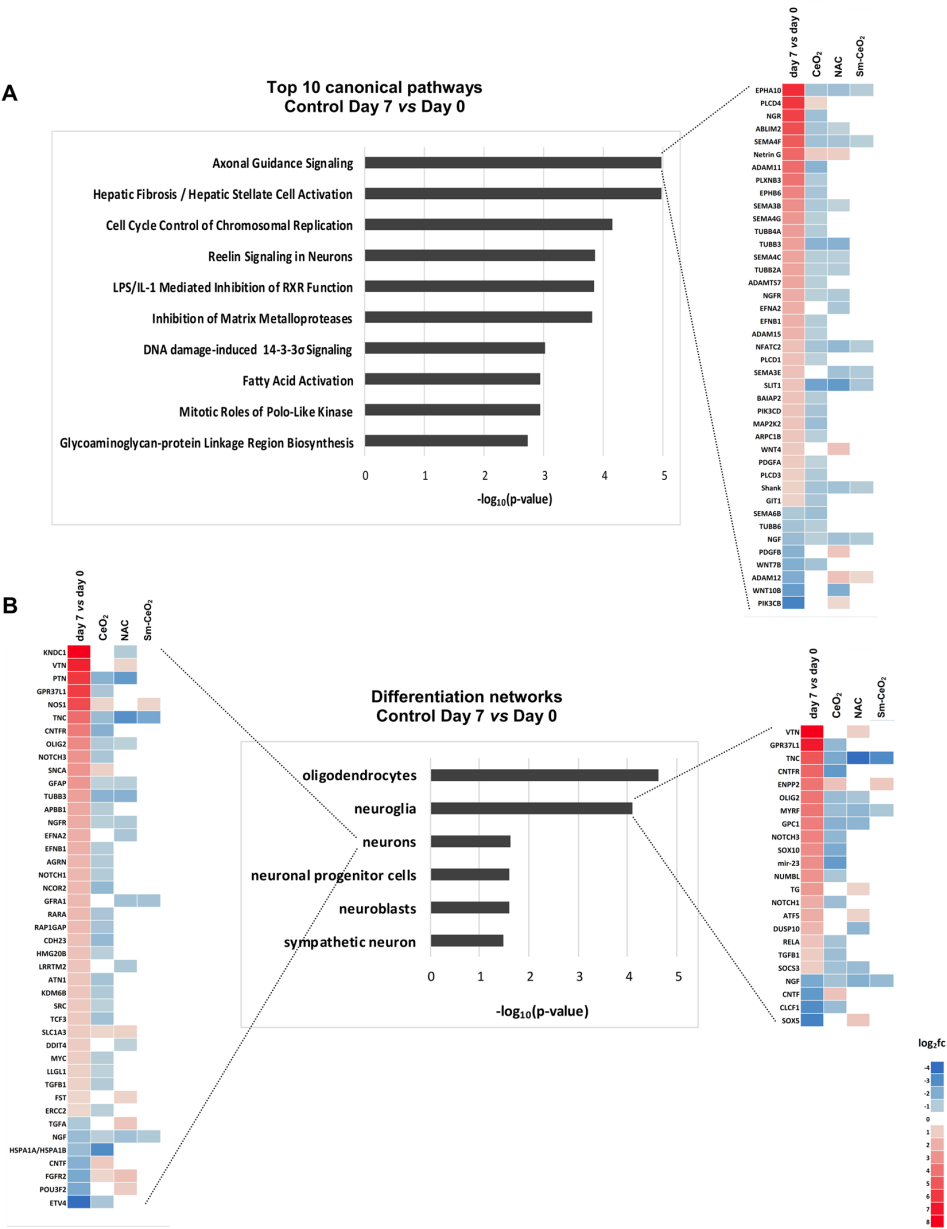
CeO_2_ impacts on axonal guidance signaling and neural stem cell differentiation. Using the IPA tool, the transcriptomics data were analyzed with respect to the most significantly perturbed pathways and networks included in the “Nervous System Development and Function” categories. (**A**) Top-10 canonical pathways relevant during neuronal differentiation of C17.2 cells (control Day 7 *versus* Control Day 0) are plotted based on their statistical significance. (**B**) Top differentiation networks from the “Nervous System Development and Function” category during neuronal differentiation of C17.2 cells (control Day 7 *versus* Control Day 0) are displayed are displayed respective to their statistical significance. Heatmaps displaying the gene expression changes in the untreated control over time were generated to show whether the genes were positively or negatively involved in the regulation of the network in this cell model. Heatmaps of the *axonal guidance signaling* pathway (**A**), or *differentiation of neurons* network and *differentiation of neuroglia* network (**B**) at day 7 are illustrated. The color coding shows the directionality of the changes in gene expression for cells treated with CeO_2_ or Sm-CeO_2_ nanoparticles, or NAC, in relation to the control. The gene lists were filtered so that they contain genes that are significantly altered in the control over time (day 7 *versus* day 0, log_2_(fold change) > 0.75) and in at least one of the treatments.

Using the Ingenuity Pathway Analysis (IPA) software tool, we investigated the pathways and the biological functions connected to the DEGs. Our main question guiding the network analysis of the transcriptomics data was related to neuronal development and differentiation. Hence, we focused the analysis on categories from “Nervous System Development and Function”. We also investigated the category “Cellular Assembly and Organization” as these processes, and the actin and microtubule cytoskeleton in particular, are important for neuronal development^52^. Notably, the canonical pathway analysis revealed that the most significantly altered pathway in the control samples (*i.e*., control day 1 *versus* day 0 and control day 7 *versus* day 0) was the *axonal guidance signaling* pathway (Fig. 5A) which is an important process for axonal extension and ultimately for the formation of a functional nervous system^53^. The relevant pathways and networks at day 1 and day 7 for all the different conditions *versus* control are summarized in Table 1, and the corresponding heatmaps are shown in Fig. 5A (day 7), and in Supplementary Figure 5 (day 1) and Supplementary Figure 6 (day 7). The results from the untreated control are also reported in order to capture the relevant pathways in C17.2 cells undergoing differentiation in the absence of particles. Due to the high number of DEGs for the control at day 7 *versus* day 0 (over 10.000 genes) we increased the log_2_ (fold change) threshold to 1.5 and the adjusted p-value threshold to 0.005 for this particular comparison to aid the biostatistical analysis. As seen in Table 1, at differentiation day 1 for the untreated samples there was a positive regulation of the networks related to “Nervous System Development and Function” such as *development of neurons*, and *neuritogenesis*, as well as networks related to “Cellular Assembly and Organization”, such as *formation of cellular protrusions*, *microtubule dynamics*, *organization of the cytoskeleton*, and the *axonal guidance signaling pathway*. Additionally, at differentiation day 7 there was a significant upregulation of *differentiation of neurons* and *differentiation of neuroglia* networks (Fig. 5B). This confirms the potential of the C17.2 cells to differentiate into a mixed cell culture under the present culture conditions^33^ and makes this a relevant model to study the effects of nanoparticles on neuronal development and differentiation. All of the generated heatmaps (Fig. 5, Supplementary Figures 5 and 6) display the gene expression changes in the untreated control over time and show whether the genes were positively or negatively involved in the regulation of the pathway/network. This serves as a benchmark for the analysis of the various treatments, and the color coding shows the directionality of the changes in gene expression, in relation to the control. In addition, the top-20 IPA canonical pathways for CeO_2_ nanoparticles *versus* control at day 1 and day 7 are summarized in Supplementary Table 1. Upon examination of the *axonal guidance signaling* pathway heatmap at day 7 (Fig. 5A), nanoceria was found to interfere with ephrin signaling (*Epha10, EphB6, EfnB1*) semaphorin signaling (*Sema4F, Sema3B, Sema4G, Sema4C, Sema6B*), neuronal growth factor signaling (*Ngf, Ngfr*) as well as with β-tubulin signaling (*Tubb4A, Tubb3, Tubb2A, Tubb6*). In all, 82.3% for CeO_2_, 80% for NAC and 87% for Sm-CeO_2_ of genes altered in this pathway had an opposite direction as compared to the untreated cells for all treatments, indicative of pathway inhibition. However, when looking at the number of considered genes, CeO_2_ had the most extensive effect (34 genes), followed by NAC (20 genes), while Sm-CeO_2_ only had a limited effect (8 genes). Next, when examining the heatmaps for the *differentiation of neurons network* and the *differentiation of neuroglia network* (Fig. 5B), we noted that expression of the genes encoding for nerve growth factor (*Ngf*) and nerve growth factor receptor (*Ngfr*), which play an important role in the neuronal development and neuroprotection^54^, was reduced in cells exposed to both nanoceria and NAC. In addition, nanoceria reduced the expression of the genes encoding for retinoid acid receptor α (*Rara*) and *Tgfβ1*, which are both known to have a positive effect on neuronal differentiation^55, 56^. In sum, 82.3% of the genes altered by CeO_2_ nanoparticles had an opposite direction compared to control for the *differentiation of neurons network* and 83.3% for the *differentiation of neuroglia network*. Thus, our computational analyses suggested that nanoceria reduces both the neuronal and neuroglial differentiation of the C17.2 neural stem cells (Fig. 5B). NAC treatment also interfered with both of these networks in a similar manner; hence, 77.8% of the genes altered in the *differentiation of neurons network* and 63.6% of the genes altered in the *differentiation of neuroglia network* had an opposite direction for NAC compared to the control. However, although NAC displayed a similar pattern as CeO_2_ nanoparticles, the effect was less extensive when considering the lower number of affected genes. The Sm-CeO_2_ nanoparticles did not alter the differentiation networks to a significant degree.

**Table 1:** Summary of next-generation sequencing results, network and pathway analysis.

Day 1	Network/Pathway name	Control Day 1 vs Day 0			CeO_2_ vs Control Day 1			NAC vs Control Day 1			Sm-CeO_2_ vs Control Day 1		
		p-value	z-score	# genes	p-value	z-score	# genes	p-value	z-score	# genes	p-value	z-score	# genes
	Development of neurons	1.97E-10	2.330	155	9.20E-10	−2.983	61	3.00E-07	2.991	48	3.89E-05	−2.518	32
	Neuritogenesis	9.87E-08	2.625	112	6.62E-10	−2.827	50	1.94E-06	2.934	37	1.28E-05	−2.415	27
	Axonal guidance signalling pathway	5.26E-15	N/A	92	2.35E-06	N/A	29	1.11E-02	N/A	17	4.51E-02	N/A	11
	Formation of cellular protrusions	9.12E-10	3.298	172	3.41E-11	−3.990	71	4.10E-05	3.026	47	1.18E-03	−2.900	31
	Microtubule dynamics	2.69E-09	2.697	214	3.97E-10	−4.202	83	2.21E-05	4.105	59	5.50E-04	−2.032	40
	Organisation of the cytoskeleton	1.20E-09	3.178	248	5.14E-11	−4.523	96	4.20E-07	4.358	73	5.56E-05	−2.374	49
**Day 7**	**Network/Pathway name**	**Control Day 7 vs Day 0**			**CeO**_**2**_ **vs Control Day 7**			**NAC vs Control Day 7**			**Sm-CeO**_**2**_ **vs Control Day 7**		
		**p-value**	**z-score**	**# genes**	**p-value**	**z-score**	**# genes**	**p-value**	**z-score**	**# genes**	**p-value**	**z-score**	**# genes**
	Development of neurons	6.99E-04	2.429	187	6.19E-07	−2.705	70	8.01E-10	−0,027	55	4.20E-05	−0.535	21
	Neuritogenesis	2.282E-05	2.282	145	4.79E-06	−2.221	53	1.90E-07	0.691	40	1.87E-05	−1.137	18
	Differentiation of neurons	2.34E-02	2.632	96	3.46E-06	−1.550	41	6.93E-05	0.359	26	NS	—	—
	Differentiation of neuroglia	7.66E-05	3.172	47	1.28E-05	−0.788	21	6.78E-06	1.628	16	NS	—	—
	Axonal guidance signalling pathway	1.05E-05	N/A	99	8.93E-08	N/A	40	9.62E-06	N/A	25	1.82E-02	N/A	8
	Formation of cellular protrusions	1.51E-03	5.503	153	5.08E-10	−2.795	88	1.53E-07	0.943	55	1.30E-04	−0.668	22
	Microtubule dynamics	9.90E-04	1.995	177	1.10E-11	−3.052	113	3.48E-07	0.409	66	3.09E-05	−0.643	28
	Organisation of the cytoskeleton	1.01E-03	1.995	178	1.91E-11	−2.658	127	6.18E-08	0.577	77	4.13E-05	−0.691	31

Murine C17.2 cells, a model of neural progenitor cells, were allowed to undergo differentiation for 1 or 7 days without exposure (control) or with CeO2, Sm- CeO2 nanoparticles or N-acetylcysteine, NAC. RNA-Seq was performed as described in the main text. Data was analysed using the IPA software with respect to enriched pathways and networks of genes. Each pathway/network is defined by the p-value, z-score and number of genes. Note that the Day 7 *vs* Day 0 analysis was based on the DEGs selected for a log2foldchange >1.5 and an adjusted p-value <0.005. All other analyses were based on DEGs selected for log2foldchange > 0.75 and an adjusted p-value <0.05. Refer to Supporting Information (Supplementary Figures 5 and 6) for heatmaps illustrating the genes involved in each pathway/network. NS, not significant; N/A, not available.

The IPA results were corroborated by Gene Ontology (GO) enrichment analysis, which was carried both on the whole DEG list at day 7 of nanoceria exposure (data not shown) as well as separately on the up- and downregulated gene sets (Supplementary Tables 3 and 4). Here, we focus the discussion on the results of the downregulated gene list as most of the genes were inhibited by nanoceria at day 7 (740 genes were downregulated out of 904 altered genes in total). Supplementary Table 5 shows the top-10 GO categories for each of the three domains (biological process, cellular component, and molecular function) ordered according to their hierarchical level, along with the corresponding genes. As seen from this summary, relevant ontologies such as *neuron projection regeneration*, *neuron projection morphogenesis, actin cytoskeleton*, and *microtubule cytoskeleton* were altered, in line with the IPA results.

Interestingly, we did not observe an enrichment in pathways related to ROS regulation which is in contradiction to other reports. For instance, in a recent study using neuron-like PC12 cells, transcription of 84 genes related with oxidative stress and antioxidant defenses was investigated using the Rat Oxidative Stress RT Profiler™ PCR Array and nanoceria was shown to downregulate the expression of some members of the glutathione peroxidase family and upregulated the gene encoding extracellular superoxide dismutase (SOD3)^57^. We therefore queried our dataset to determine whether the expression of any of the genes from this oxidative stress array was altered. At day 1, NAC altered the gene expression of 9 genes (*Aox1, Gclc, Gclm, Hmox1, Hspa1A/Hspa1B, Ncf2, NQO1, Ptgs2, Srxn1*), suggesting interference with the oxidative stress balance, but with no effect from the particles. At day 7, the only gene altered was *Nos2*, which was downregulated both by CeO_2_ and NAC. Overall, it seems the effects of nanoceria on the oxidative balance as defined by “the rat oxidative stress profiler” are subtle, and not fully overlapping with the antioxidant effects of NAC. Moreover, previous microarray studies reported that nanoceria can be neurotoxic by reducing huntingtin (*Htt)* gene expression and activity of related pathways with induction of cell cycle arrest and apoptosis in the HT22 hippocampal nerve cell line^58^. However, no such effects were observed in the current cell model.

In conclusion, based on the transcriptomics data, both nanoceria and NAC are predicted to inhibit neuronal and neuroglial differentiation and axonal guidance signaling in C17.2 neural stem cells, while Sm-doped particles appear less effective, arguing in favor of an antioxidant effect. We therefore hypothesized that CeO_2_ nanoparticles suppress neuronal differentiation in an antioxidant-dependent manner and we designed validation experiments as described below.

### CeO_2_ nanoparticles suppress neural and neuroglial stem cell differentiation

**Figure 6:**
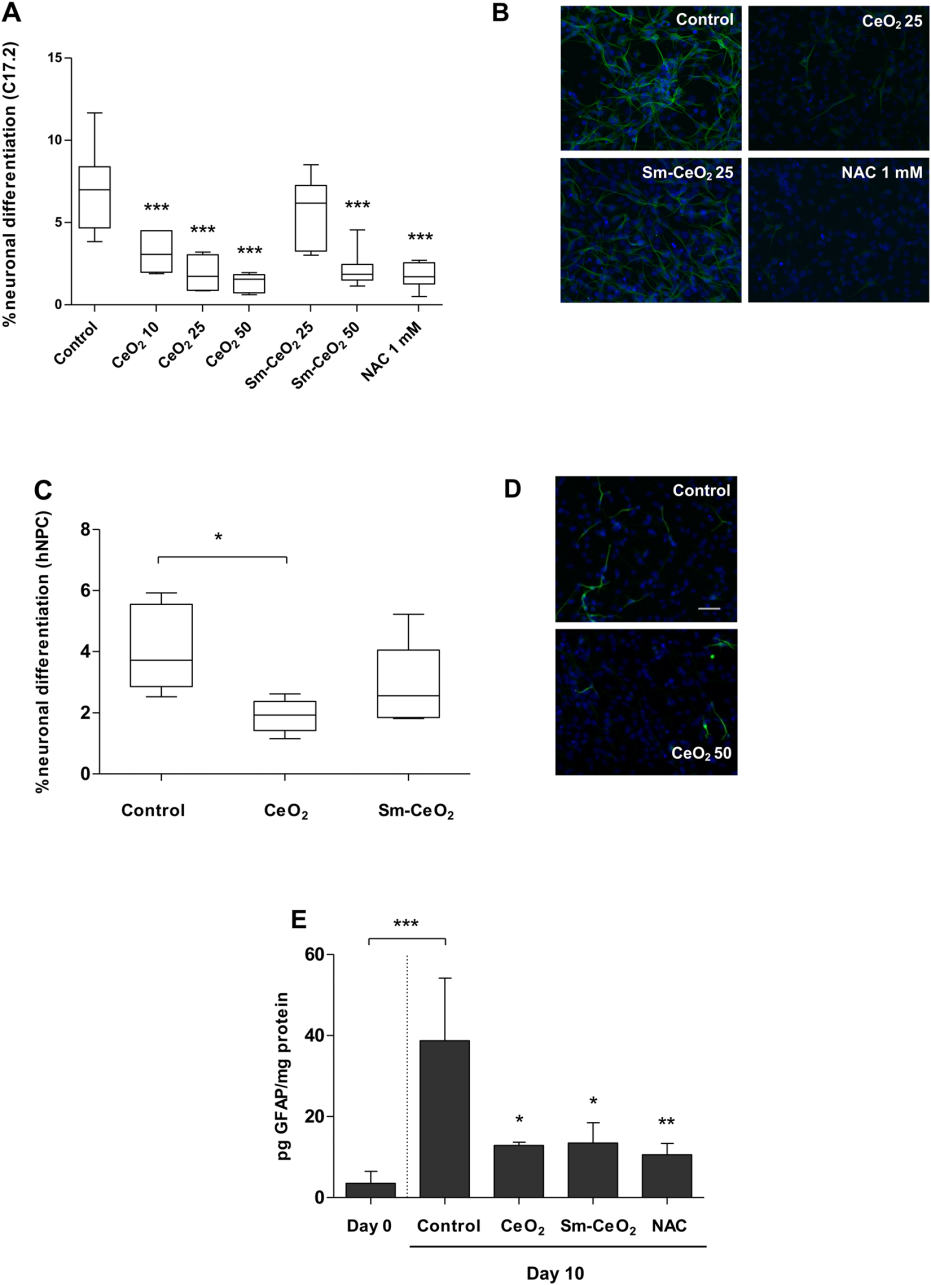
CeO_2_ suppresses neural differentiation of murine and human stem/progenitor cells. C17.2 cells were differentiated for 7 days in the presence of CeO_2_ (10, 25, 50 μg/mL), Sm-CeO_2_ (25, 50 μg/mL) or NAC (1 mM). After exposure, cells were fixed and stained for β3-tubulin (TuJ1 antibody, green) and DNA (DAPI, blue). The slides were scored for the number of TuJ1 positive cells (considering fluorescence intensity and cellular morphology) (**A**). Results are presented as box plots of at least 6 replicates (the box corresponds to the interquartile range, the line inside the box is plotted at the median and the whiskers show the minimum and maximum values). Significant results are marked with asterisks (***p-value <0.001). (**B**) Representative images using an inverted fluorescence microscope with a 20X objective. Human neural progenitor cells (hNPC) were differentiated for 4 days in the presence of CeO_2_ or Sm-CeO_2_ (50 μg/mL). Cells were stained for β3-tubulin (mS58 antibody, green) and DNA (DAPI, blue) and slides were scored for the number of β3-tubulin positive cells (taking into account fluorescence intensity as well as cellular morphology) (**C**). Results are presented as box plots of 6 replicates (each box corresponds to the interquartile range, the line inside the box is plotted at the median and the whiskers show the minimum and maximum values). Significant results are marked with asterisks (*for p-value <0.05). (**D**) Representative images (20X). (**E**) Expression of GFAP. C17.2 cells were treated with CeO_2_ (25 µg/mL), Sm-CeO_2_ (25 µg/mL) or NAC (1 mM) and differentiated for 10 days. ELISA was performed on cell lysates and results were normalized according to protein content. Results are shown as mean values ± S.D. (n = 3). Significant results are indicated with asterisks (*p-value <0.05, **p-value <0.01).

To validate the RNA-Seq findings related to neuronal differentiation, we evaluated the expression of neuron specific β3-tubulin (TuJ1) as a marker of neuronal differentiation^59^ by immunostaining. The results showed that CeO_2_ nanoparticles significantly and dose-dependently reduced the number of TuJ1 expressing cells (Fig. 6A,B). NAC also suppressed neuronal differentiation in this model, as evidenced by TuJ1 staining. No effect was seen for the Sm-CeO_2_ nanoparticles at 25 μg/mL, while a significant effect on the number of TuJ1 expressing cells was noted at the high dose (50 μg/mL). This could be explained by the fact that the Sm-doped CeO_2_ still retain some residual antioxidant activity^20^, which may be sufficient to alter the redox balance. To assess whether nanoceria exerted direct, cytotoxic effects towards the newly formed neurons, thereby skewing the results, we tested the toxicity of nanoceria in differentiated C17.2 cells. CeO_2_ particles was non-toxic to the differentiated C17.2 cells up to 100 μg/mL (Supplementary Figure 7).

To corroborate the findings obtained using the murine C17.2 cell line, we employed primary human neural progenitor cells (hNPC). The cells were differentiated for 4 days in the presence of CeO_2_ or Sm-CeO_2_ (50 μg/mL). Cells were then fixed and stained for β3-tubulin (TuJ1). The results showed that the CeO_2_ nanoparticles, but not the Sm-CeO_2_ nanoparticles, were capable of suppressing hNPC differentiation (Fig. 6C,D) which is consistent with our C17.2 experiments, and suggestive of an antioxidant effect. In light of the fact that our transcriptomics results revealed effects not only on the *differentiation of neurons*, but also on the *differentiation of neuroglia* networks, we decided to address whether CeO_2_ or Sm-CeO_2_ nanoparticles also would affect neuroglial differentiation. To this end, we selected GFAP (glial fibrillary acidic protein) as a marker of neuroglial cells, more specifically of astrocytes^60^. Quantification of GFAP was performed by ELISA following 10 days of differentiation. The experiment was prolonged to 10 days since we did not observe a significant increase in GFAP expression in control cells after 7 days of differentiation (data not shown). As seen in Fig. 6E, both CeO_2_ and Sm-CeO_2_ nanoparticles (25 µg/mL) significantly decreased the expression of GFAP. The same effect was noted for NAC (1 mM). These results lend some support to the RNA-Seq based predictions, but it is noted that the RNA-Seq analysis was conducted at day 7, not 10 days. Taken together, we show for the first time that nanoceria inhibits neural stem cell differentiation.

The possibility that nanoceria could interfere with cell differentiation has been addressed in previous studies, albeit with divergent outcomes. Hence, nanoceria did not impair the ability of murine cardiac progenitor cells to differentiate into adipogenic, osteoblastic, or cardiac lineages^12^. In contrast, other investigators have reported that nanoceria inhibited adipogenic differentiation of rat mesenchymal stem cells as a function of its antioxidant activity^61^. Nanoceria was also shown to increase neurite length indicative of neuronal differentiation along with dopamine secretion in PC12 cells^62^. The reported differences could be explained by the different models used (*i.e*., mouse or human neural stem cells *versus* a rat pheochromocytoma cell line) as well as different nanoparticles with varying physicochemical properties. In addition, differentiation of neural and cardiac cells may be differently regulated. It is important to note that all cellular processes related to neural differentiation – embryonic or adult stem cell proliferation and differentiation, *in vitro* generation of neurons from progenitor cells, and the development of neuroblastomas – are tightly controlled by oxygen concentrations^44^.

### Further probing of antioxidant *versus* particle effects of nanoceria

The RNA-Seq downstream analysis suggested some similarities between the two nanoparticles, CeO_2_ and Sm-CeO_2_, that were not shared with NAC, namely inhibition of “Cellular Assembly and Organization” networks. Thus, both at day 1 and day 7, the CeO_2_ nanoparticles induced a significant suppression of the *organization of the cytoskeleton*, *microtubule dynamics*, and *organization of the cytoplasm* networks (Table 1, and see heatmaps in Supplementary Figures 5 and 6). The Sm-CeO_2_ nanoparticles showed a similar pattern, albeit not as pronounced at day 7. On the other hand, NAC had a very different effect on the three networks related to “Cellular Assembly and Organization”. Hence, both at day 1 and day 7, NAC activated the *organization of the cytoskeleton*, *microtubule dynamics* and *organization of the cytoplasm* networks, with a stronger effect at the earliest time-point, day 1 (Table 1, and see heatmaps in Supplementary Figures 5 and 6). To specifically address the differences between nanoceria *versus* the conventional antioxidant NAC, we examined the top-20 canonical pathways for CeO_2_ *versus* NAC at day 1 and day 7 (Supplementary Table 2). Among the top altered pathways were the *actin cytoskeleton signaling*, *axonal guidance signaling*, and *integrin signaling* pathways. These pathways are interconnected and relevant for neuronal development and function^63^. Integrins are cell anchoring receptors that mediate interactions with the extracellular matrix to coordinate axonal guidance and outgrowth^63^. The integrin signaling pathway was significantly inhibited by CeO_2_, with downregulation of *ItgA3, ItgA4, ItgA7, ItgA2B, ItgB5* (Supplementary Table 2). Nanoparticles could impact on cytoskeletal dynamics and organization directly, from within the cytoplasm, or indirectly through interactions with extracellular matrix components or transmembrane receptors (*e.g*., integrins). Interestingly, a recent study has shown that sublethal concentrations of silver nanoparticles disrupted cytoskeletal organization and neurite extension in cultured adult neural stem cells derived from rat brain, likely through a direct effect on actin dynamics^64^. In contrast, our results suggested an effect of nanoceria at the transcriptional level in differentiating neural stem cells. We also examined the GO enrichment of the genes that were differentially expressed and shared by CeO_2_ and Sm-CeO_2_ at day 1 (107 genes) and at day 7 (30 genes). The low number of genes at day 7 precluded further analysis. The results at day 1 showed involvement of ontologies such as *integrin-mediated signaling*, *integrin complex* and *integrin binding* defined by *Itga1*, *Itga2, Itgb2* and *Gpnmb* genes (Supplementary Table 5). This is consistent with the notion that interference with integrin signaling is a ‘particle effect’ that is not related to the antioxidant function of nanoceria. Further studies on integrin signaling and its relation to cytoskeletal organization in cells exposed to nanoceria *versus* conventional antioxidants are warranted.

### Super-resolution microscopy reveals particle effects on the neural growth cone

**Figure 7:**
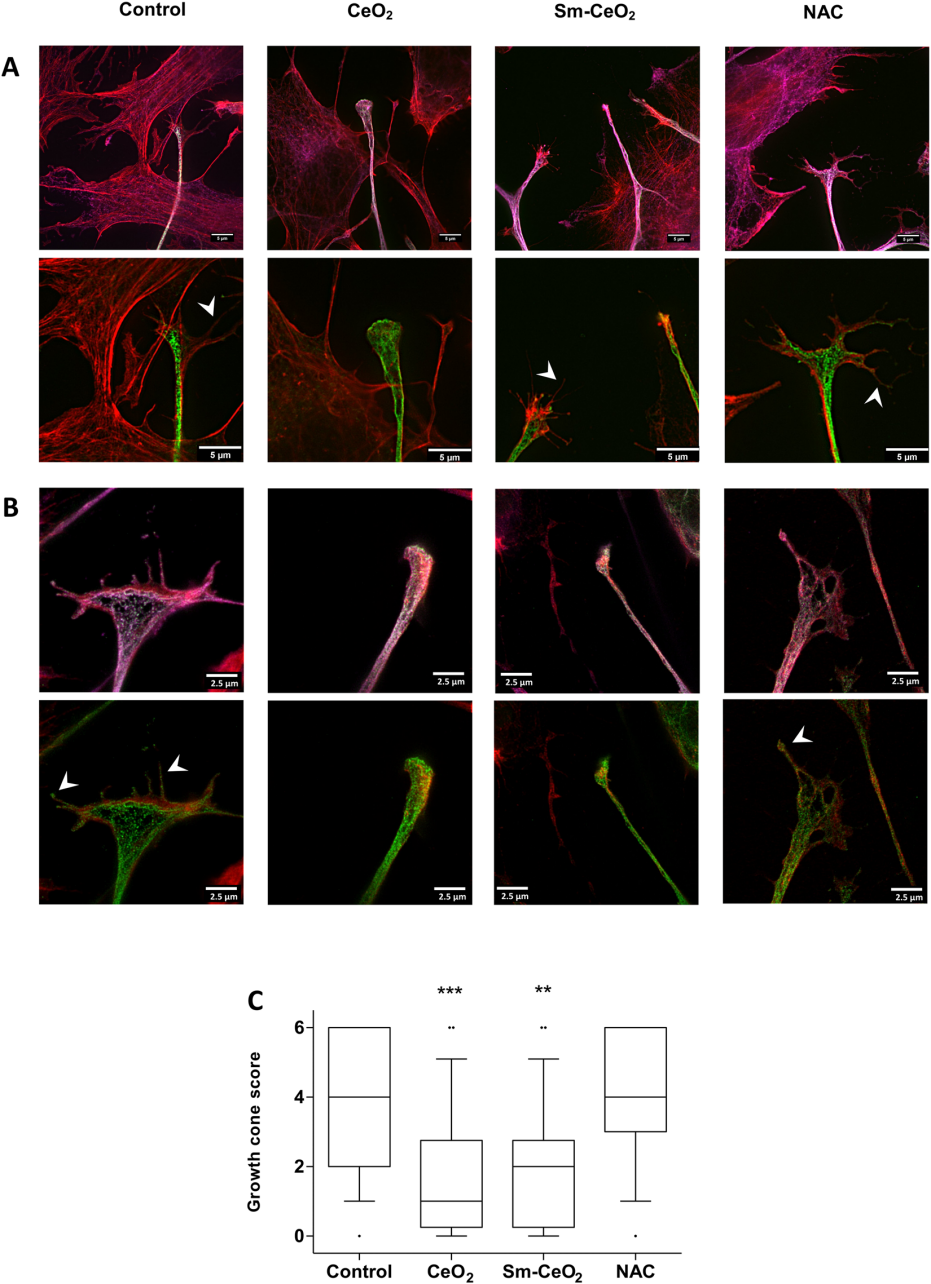
CeO_2_ and Sm-CeO_2_ nanoparticles alter the structure of neural growth cones. The structure of the growth cones was investigated by using super-resolution microscopy. C17.2 cells were differentiated for 6 days in the presence of CeO_2_ (25 μg/mL), Sm-CeO_2_ (25 μg/mL) or NAC (1 mM). After exposure, cells were fixed and stained for tyrosinated tubulin (ABT171 antibody, magenta) and F-actin (phalloidin-TRITC, red) [upper row of images in (**A**) and (**B**)] or β3-tubulin (anti-TuJ1 antibody, green) and F-actin (phalloidin-TRITC, red) [lower row of images in (**A**) and (**B**)]. Representative images taken using SIM (**A**) and STED (**B**) microscopy are shown and filopodia are indicated by white arrowheads. Note that the lower row of images in (**A**) represents a higher magnification of the images shown in the upper row. (**C**) Approx. 30 growth cones per condition were scored for number of filopodia and morphology as described in Methods. Results are presented as box plots (the box corresponds to the interquartile range, the line inside the box is plotted at the median and the whiskers show the 10–90 percentiles). Significant results are marked with asterisks (**p-value < 0.01, ***p-value < 0.001).

The results obtained above suggested a particle effect, shared by both CeO_2_ and Sm-CeO_2_, but not related to or overlapping with the activity of NAC. It remains possible, however, that nanoceria exerts additional antioxidant effects not displayed by NAC. Indeed, NAC is a metabolic antioxidant that acts by replenishing the cysteine pool that ultimately leads to glutathione synthesis^65^. Nanoceria, on the other hand, acts as a direct scavenger of ROS and possesses an enzyme mimetic function similar to superoxide dismutase (SOD) and catalase^66^. In order to test the ‘particle effect’ hypothesis, and probe for interactions of the CeO_2_ and Sm-CeO_2_ nanoparticles with cytoskeleton related networks (*formation of cellular protrusions*, *microtubule dynamics* and *organization of the cytoskeleton*) we performed experiments focusing on neural growth cones in differentiating C17.2 cells. We first asked whether exposure to nanoceria affected the expression of endogenous antioxidant proteins as this could confound the results. However, as seen in Supplementary Figure 8, CeO_2_ (25 µg/mL), Sm-CeO_2_ (25 µg/mL) did not alter protein expression of mitochondrial SOD (SOD2) (A) or catalase (B) in C17.2 cells. NAC (1 mM) decreased the expression of SOD2, but did not affect the expression of catalase. Neural growth cones are dynamic structures at the distal tip of a developing neurite which are involve in axonal guidance and pathfinding, a process by which neurons connect with appropriate synaptic partners during embryogenesis^67^. During axonal guidance, the growth cones are crucial for translating guidance cues to rearrangements of the cytoskeletal structure^68^. Growth cone dynamics is driven by cyclical polymerization and depolymerization of actin filaments, which in turn determine the formation of growth cone protrusions (filopodia and lamellipodia)^68^. In order to visualize the neural growth cones we used two super-resolution microscopy techniques, structured illumination microscopy (SIM) together with stimulated emission depletion (STED) microscopy, and we imaged three cytoskeletal proteins (tyrosinated tubulin, TuJ1 and F-actin). Tuj1 (β3-tubulin) is a marker for neuronal cells (see Fig. 6) while tyrosinated tubulin is found in the axonal shaft and the growth cone; actin filaments (F-actin) are enriched in the peripheral domain of the growth cone and microtubules are located in the central domain and interact with the actin filaments^52^. The fine extensions of the growth cone are known as filopodia^69^. To quantify the results, the imaged growth cones were scored on the basis of their morphology and number of filopodia, as described in Methods. As shown in Fig. 7A,B, the growth cones were smaller, and less likely to display the typical triangular structure following exposure of cells to CeO_2_ and Sm-CeO_2_. NAC, on the other hand, did not appear to affect the growth cone structure (see Fig. 7C for a semi-quantitative analysis of the results). Single channel images of the three probes (tyrosinated tubulin, F-actin, TuJ1) are shown in Supplementary Figure 9 (SIM) and Supplementary Figure 10 (STED). In conclusion, both CeO_2_ and Sm-CeO_2_ interfered with the neural growth cone morphology in differentiating C17.2 cells, suggestive of a particle effect. These results are consistent with the RNA-Seq analysis.

## Conclusions

Nanoceria was suggested in previous reports to exert effects on primary neural cells or cell lines, and in some cases, gene expression studies were conducted to shed light on the underlying mechanisms^58, 62^. However, the present study is the first to our knowledge to explore the potential impact of nanoceria on neural progenitor cells. In this study, we have used next-generation sequencing approaches as well as conventional biological assays and super-resolution microscopy techniques to study the effects of nanoceria in the murine cell line C17.2, a commonly used model of developmental neural stem cells^70^. Sm-doped nanoceria was included in order to evaluate antioxidant effects, along with the antioxidant, NAC. We demonstrated that nanoceria was non-cytotoxic for neural progenitor cells and, moreover, displayed a transient cytoprotective effect by suppressing ROS generation induced by the prooxidant, DMNQ. On the other hand, our results revealed that nanoceria inhibited neural stem cell differentiation, at least in part due to its antioxidant properties, as these effects were seen when cells were exposed to CeO_2_, but not in response to Sm-CeO_2_. The inhibition of neural stem cell differentiation was confirmed in murine C17.2 cells and primary human neural progenitor cells (hNPC). In addition, RNA-Seq also predicted a potential particle effect (for both particles) on cellular assembly networks. This hypothesis was tested by super-resolution imaging of neural growth cones and the results confirmed that both nanoparticles, but not NAC, interfered with the neural growth cone. One may conclude that the latter effects are not “NAC-like”, but the effects could still be associated with a (residual) antioxidant effect. There is emerging evidence that the organization as well as the dynamics of the cytoskeleton could also be regulated by the redox balance during neuronal development^69^ and it cannot be completely ruled out that the antioxidant effect of the particles contributed to the observed effects. Sm-doped CeO_2_ nanoparticles are known to retain a small antioxidant effect^20^ and one may postulate that this is sufficient for disruption of the delicate redox balance in neural progenitor cells.

Taken together, we have provided evidence that nanoceria acts as a double-edged sword and our findings have underscored that antioxidant properties are not necessarily beneficial insofar as ROS production is not always detrimental to the cell. On the contrary, ROS are critically involved in neuronal differentiation^44^. Moreover, the current study has demonstrated the utility of RNA-Seq coupled with computational analysis of the transcriptomics data in unraveling the impact of nanoceria on neuronal differentiation, as well as the potential particle *versus* antioxidant (or, NAC-like) effect of nanoceria, which may not be as evident by conventional approaches. The current study was performed using the murine C17.2 neural progenitor cell line, a commonly used model in developmental neurobiology^71^ and neurotoxicology^70^ research. However, it is important to acknowledge that all *in vitro* models may have advantages and disadvantages^72^, and further studies using other models are needed to support the current results. Nevertheless, we believe that our findings are relevant both for the potential biomedical applications of nanoceria and for the inadvertent occupational or environmental exposure to nanoceria and that particular care should be taken with respect to the developing nervous system.

## Materials and Methods

### Nanomaterials

The CeO_2_ nanoparticles and the 20% Samarium (Sm) doped CeO_2_ nanoparticles (Sm-CeO_2_) were synthesized by a wet chemical process at room temperature as previously described^20, 35^. The nanoparticle suspensions were prepared in MilliQ water at a stock concentration of 20 mg/mL by sonication for 5 min at 10 amplitude microns in an ultrasonic disintegrator running with an exponential probe (Soniprep 150, MSE). New stock dispersions were prepared before each experiment. In order to reach the indicated exposure concentrations, the stock dispersion was diluted in the relevant cell medium prior to exposure.

### Nanomaterial characterization

Detailed characterization of the physicochemical and antioxidant properties of the CeO_2_ and Sm-CeO_2_ nanoparticles is described elsewhere^20^. Here, primary particle size as well as hydrodynamic size and surface charge was determined (Supplementary Figure 1). High resolution transmission electron microscopy images were taken by using a 300 keV electron beam energy by using a Titan G_2_ 60–300 ST Cs-Image corrected microscope and the primary particle size was estimated to be approx. 10 nm for CeO_2_ and approx. 13 nm for Sm-CeO_2_^20^. Additionally, the nanoparticles were characterized in cell medium by dynamic light scattering (DLS). The hydrodynamic diameter of the nanoparticles at different concentrations (20–100 µg/mL) was measured at 37 °C in C17.2 medium and differentiation media, as well as milliQ water (pH adjusted to 7.4). Measurements were performed using a Malvern Zetasizer (Nano-ZS, Malvern Instruments, Worcestershire, UK) using disposable cuvettes. DLS experiments consisted of 15 runs per measurement of 10 s each. All experiments were carried out in triplicate. The mean values ± S.D. of the hydrodynamic diameter and the polydispersity index (P.I.) are reported. Zeta potential was measured at a concentration of 50 µg/mL at 37 °C in milliQ water (pH = 7.4) and in the two culture media. Experiments were carried out in triplicate and diameters were reported as mean value ± S.D. The measurements in milliQ H_2_O were made in general purpose mode at 150 V; the measurements in cells media were collected in monomodal mode at low voltages, due to the high conductivity of this sample (16 mS/cm).

### Cells and cell culture conditions

C17.2 cells are mouse derived neural progenitor cells reported to be a relevant *in vitro* model for testing developmental neurotoxicity^34^. Under proliferating conditions the C17.2 cells were grown in cell culture dishes in DMEM high glucose, GlutaMAX (Gibco) supplemented with 5% horse serum, heat inactivated (Gibco) and 10% fetal bovine serum, heat inactivated (Gibco), 2 mM glutamine (Gibco), 100 U penicillin/mL, and 100 U streptomycin/mL (Gibco). The cells were split every 3–4 days. For differentiation experiments the cells were seeded at a density of 2000 cells/cm^2^ in suitable cell culture plates in DMEM complete cell medium. After 24 h the cell medium was replaced with differentiation medium consisting of DMEM:F12 medium (Gibco) supplemented with 2 mM glutamine (Gibco), 100 U penicillin/mL, 100 U streptomycin/mL (Gibco) and N2 supplement (1:100) (ThermoFischer Scientific). Half of this differentiation medium was changed every 3 days during differentiation. The cells were incubated in a humidified atmosphere at 37 °C, 5% CO_2_ for all experiments.

In addition, primary hNPC cells isolated from the subcortical forebrain region of terminated first trimester embryos were used for differentiation experiments (ethical committee approval no 2013/564-32, Regional Ethical Committee, Stockholm). The hNPC were kindly provided by Dr. Erik Sundström, Department of Neurobiology, Care Sciences and Society, Karolinska Institutet. Informed consent was obtained from all participants as stipulated by the ethical committee and all experiments were conducted in accordance with relevant guidelines. The cells were subcultured as neurospheres in defined serum free media composed of Dulbecco’s Modified Eagle’s Medium and Ham’s F12 (3:1) supplemented with B27 (Invitrogen), 20 ng/ml epidermal growth factor (EGF) (Invitrogen), and 20 ng/ml recombinant human fibroblast growth factor (rhFGF) (R&D Systems) at 37 °C with 5% CO_2_ as previously described^73^. Passaging was performed mechanically using a McIlwain tissue chopper. For differentiation experiments, neurospheres were dissociated to a single cells suspension and then plated on poly-d-lysin/laminin coated coverslips and allowed to attach in differentiation medium consisting of DMEM:F12 medium with N2 supplement, but without EGF and FGF.

### Cell viability assay

Cell viability of the of the proliferating C17.2 cells after nanoparticle exposure was assessed by the Alamar Blue assay (ThermoFischer Scientific). C17.2 cells were seeded in 48-well plates at a density of 6000 cells/cm^2^ in complete DMEM medium. The following day the medium was carefully removed and the cells were exposed to the same medium containing 5, 10, 20, 50, 100 µg/mL CeO_2_ or Sm-CeO_2_ nanoparticles for 48 h. At the end of the exposure the medium was changed to medium containing 10% Alamar Blue and further incubated for 2 h. The fluorescence was measured at 560 nm excitation and 590 nm emission wavelength using a plate reader (Tecan Infinite F200, Tecan Trading AG, Switzerland). Results were expressed as % cell viability compared to the untreated control.

### Cellular ROS generation

The generation of cellular ROS following CeO_2_ and Sm-CeO_2_ exposure alone and in the presence of an oxidative insult was investigated by the dichloro-dihydro-fluorescein diacetate assay (DCFH-DA). Briefly, C17.2 cells were seeded at a density of 6000 cells/cm^2^ in 96-well plates with transparent bottom. The following day the cells were incubated with CeO_2_ (20, 50, 100 µg/mL) or Sm-CeO_2_ (20, 50, 100 µg/mL) NPs for 4 h and then loaded with DCFH-DA probe (25 µM, 45 min). Next cells were incubated with fresh medium (phenol red-free) or medium containing the pro-oxidant, dimethoxy-naphthoquinone (DMNQ, 10 μM) (Sigma-Aldrich). ROS formation was assessed on a plate reader (excitation 485 nm, emission 535 nm) at the indicated time-points. ROS induction was expressed as fold change *versus* control.

### Caspase-3/-7 activity assay

Caspase activity was determined by incubation of cell lysates with the fluorogenic substrate DEVD-AMC (50 µM) (Sigma-Aldrich) as previously described^74^, and the enzyme catalyzed release of AMC was quantified using a plate reader (Tecan Infinite F200) (excitation 360 nm, emission 465 nm) in a kinetic mode over 45 min with readings performed every 90 s at 37 °C. Caspase-3/-7 activity was determined from the slope of the released AMC and expressed as fold change *versus* untreated control. Etoposide (Sigma-Aldrich) was included as a positive control (100 µM, exposure 4 h before cell harvesting).

### Automated microscopic morphological assessment

Cell viability following oxidative insult was monitored by microscopic assessment of cell morphology and cell numbers using a Cell-IQ 2 automated live cell imaging platform (Chip-Man Technologies Ltd). Briefly, C17.2 cells were seeded at a density of 2000 cells/cm^2^ in 48-well plates two days in advance. C17.2 cells were exposed to CeO_2_ and Sm-CeO_2_ nanoparticles (20, 50 μg/mL) for 4 h, then challenged with DMNQ (10 μM). The plate was placed in the Cell-IQ 2 instrument, and light microscopy images (phase contrast) were taken (using the Imagen software) at two defined locations in each well every 30 min continuously over 12 h. The images taken at the indicated time-points were manually scored in terms of number of non-dividing flat cells, mitotic cells and dying/dead cells. Results were expressed as % increased cell death after 8 h and 12 h as compared to time-point 0 h.

### Transmission electron microscopy

Cellular uptake and intracellular localization of the nanoparticles were visualized by TEM. Proliferating cells C17.2 cells were seeded in 6-well plates at a density of 6000 cells/cm^2^ in complete DMEM medium. The following day the medium was carefully removed and the cells were exposed to medium containing 50 µg/mL CeO_2_ or 50 µg/mL Sm-CeO_2_ for 24 h. Alternatively, proliferating cells C17.2 cells were seeded in 6-well plates at a density of 2000 cells/cm^2^ in complete DMEM medium and the medium was carefully removed the following day and the cells were exposed to differentiation medium containing 50 µg/mL CeO_2_ or 50 µg/mL Sm- CeO_2_ for 7 days, changing half of the differentiation medium at day 3 and at day 6. At the end of the exposure the cells were washed, harvested by trypsinization and fixed in freshly prepared 2.5% glutaraldehyde in 0.1 M phosphate buffer (PB). After fixation the pellet was rinsed in 0.1 M PB and post fixed in 2% osmium tetroxide in 0.1 M PB, pH 7.4 at 4 °C for 2 h, dehydrated in ethanol followed by acetone, and embedded in LX-112 (Ladd, Burlington, Vermont, USA). Ultrathin sections (approximately 60–80 nm) were cut by a Leica ultracut UCT (Leica, Wien, Austria) and contrasted with uranyl acetate followed by lead citrate and examined with Tecnai 12 Spirit Bio TWIN transmission electron microscope (Fei company, Eindhoven, The Netherlands) at 100 kV. Digital images were captured by using a Veleta camera (Olympus Soft Imaging Solutions, GmbH).

### Inductively coupled plasma mass spectrometry

Cellular uptake of the CeO_2_ and Sm-CeO_2_ nanoparticles was quantified using inductively coupled plasma mass spectrometry (ICP-MS). C17.2 cells were seeded in 6-well plates at a density of 6000 cells/cm^2^ in complete DMEM medium. The following day the medium was carefully removed and the cells were exposed to medium containing 10, 25, 50 µg/mL CeO_2_ or Sm-CeO_2_. Alternatively, C17.2 cells were seeded in 6 well plates at a density of 2000 cells/cm^2^ in complete DMEM medium and the medium was carefully removed the following day and the cells were exposed to differentiation medium containing 25 µg/mL CeO_2_ or 25 µg/mL Sm-CeO_2_ for 1 or 7 days. At the end of the exposure the cells were washed three times with PBS, harvested by trypsinization, resuspended in cell medium and counted. The mineralization of the samples was performed in 45% HNO_3_ for 7 days. Thereafter the samples were diluted to reach a 2% HNO_3_ concentration and ^140^Ce, ^142^Ce, ^147^Sm, ^149^Sm, ^152^Sm, ^154^Sm isotopes were quantified using an iCAP Q ICP-MS (Thermoscientific) instrument running on KED mode. Matrix matched calibration standards of Ce and Sm (0.1, 1, 5, 10, 50, 100, 500 ppb) were prepared using untreated control samples to account for the complexity of matrix due to the cell debris and medium components. All samples were spiked with 5 ppb In, as an internal standard. The range for internal standard recovery was between 90–110%. The limits of detection for the investigated isotopes were 0.019 (^140^Ce), 0.09 (^142^Ce), 0.007 (^147^Sm), 0.029 (^149^Sm), 0.009 (^152^Sm) and 0.002 (^152^Sm) ppm. Each sample was injected 6 times and the RSD acceptance was set at 20%. Results were normalized according to the cell count and expressed as pg Ce and Sm/cell considering the average values.

### Immunocytochemistry

Differentiation of C17.2 and hNPC cells into neurons was quantified using immunocytochemical stainings of β3-tubulin (TuJ1). C17.2 cells were seeded at a density of 2000 cells/cm^2^ on glass coverslips in 24-well plates in complete DMEM medium. The following day the cell medium was changed to differentiation medium alone or containing CeO_2_ (10, 25, 50 μg/mL), Sm-CeO_2_ (25, 50 μg/mL), or NAC (1 mM). The C17.2 cells were differentiated for 7 days and at the end of the exposure the cells were washed and fixed in 4% formaldehyde for 20 min. hNPC cells were seeded at a density of 20.000 cells/cm^2^, exposed to 50 μg/mL CeO_2_ or 50 μg/mL Sm-CeO_2_ or left unexposed (control), and differentiated for 4 days. The choice of time-point for the hNSC was based on technical considerations that made it easier to score the differentiated neurons. Thereafter the coverslips were washed in PBS and blocked/permeabilised in 0.3% Triton-X (Sigma-Aldrich) with 10% donkey serum (Jackson ImmunoResearch) for 30 min. Then the coverslips were incubated with mouse anti-β3-tubulin (TuJ1) antibody (Covance) prepared in the same blocking solution overnight at 4 °C. The coverslips were then rinsed in PBS and incubated with DAPI and secondary antibody, donkey anti mouse antibody 488 Alexa conjugated (Jackson ImmunoResearch) for 1 h. The coverslips were mounted on microscopy slides using mounting medium (Dako, Agilent Technologies) and pictures were taken using an inverted Nikon ECLIPSE TE2000-S fluorescence microscope (Nikon Corp., Tokyo, Japan). At least 6 fields were considered per slide and at least 6 slides were scored per exposure. The slides were blindly scored in terms of number of neurons (considering the TuJ1 intensity and cell morphology). The results were expressed as % neuronal differentiation based on the percentage of TuJ1 positive cells.

### Enzyme-linked immunosorbent assay (ELISA)

C17.2 cells were differentiated for and 7 days (10 days for GFAP) under continuous exposure to CeO_2_ (25 μg/mL), Sm-CeO_2_ (25 μg/mL) or NAC (1 mM). Quantification of cellular expression of GFAP (glial fibrillary acidic protein), SOD2 (superoxide dismutase, mitochondrial), or catalase was determined on cell lysates using ELISA kits (GFAP - EKM670, SOD2 - EKM2744, CAT - EKM714; NordicBiosite) according to the manufacturers’ protocol. Results were normalized according to the protein content and expressed as quantity of the protein of interest per mg total protein.

### Super-resolution microscopy

C17.2 cells were seeded on poly-d-lysin/laminin-coated coverslips and exposed and differentiated as described for the immunocytochemistry protocol. The cells were differentiated for 6 days in order to reach an optimal cell density suitable for imaging. Thereafter, the cells were fixed 2% formaldehyde, washed in PBS and blocked/permeabilised in 0.1% Triton-X (Sigma-Aldrich) with 2% bovine serum albumin (Sigma) for 1 h. Then the coverslips were incubated with mouse anti-β3-tubulin (TuJ1) antibody (Covance) and rabbit anti-tyrosinated tubulin (ABT171) antibody (Abcam) prepared in the same blocking solution overnight at 4 °C. The coverslips were then rinsed in PBS and incubated with DAPI (4′,6-diamidino-2-phenylindole) (to visualize cell nuclei), phalloidin-TRITC (tetramethylrhodamine) (high affinity probe for F-actin) as well as secondary antibodies, donkey anti mouse antibody 488 Alexa conjugated (Jackson ImmunoResearch) and goat anti rabbit STAR 635 P (Abberior) for 1 h. The coverslips were then rinsed in PBS and mounted on microscopy slides using ProLong Gold mounting medium (ThermoFischer). Super-resolution images were taken on a SIM microscope (Zeiss Elyra PS.1) and STED microscope (Leica SP8 STED). Approx. 30 mature growth cones per condition were scored blindly according to their morphology (0 – cylindrical, 1 – round/oval, 2 – small triangular, 3–triangular shape) as well as number of filopodia (0−0–2, 1 −>3, 2 −>5, 3 −>7). SIM imaging was done with 405-, 488-, 561- and 642-nm excitation lasers, using a Plan-Apochromate 100x/1.46 NA oil lens. Emission was collected sequentially through appropriate dichroic mirrors and bandpass filters set at 420–480 nm for 405 nm excitation, 495–575 nm for 488 nm excitation, 570–650 nm for 561 nm excitation, and above 655 nm for 642 nm excitation onto a EMCCD camera (iXon 897, Andor Technology). Camera gain was set to 15–25 with camera integration times between 80 to 250 ms per imaged channel. SIM processing was done with the included ZEN software 2012 (SP2), with selection of automatic settings for evaluation of the raw data (*i.e*. theoretical PSF, selection of noise filter setting, frequency weighting, baseline settings etc.)^75^. The optimal grid size was automatically assigned to each wavelength by the software, and the grid was rotated 5 times at 5 phases for each image. Calibration on 40 nm beads generated a lateral precision of 81 nm ± 5 nm at 488 nm.

To confirm the SIM generated images, super-resolution STED imaging was applied, using a Leica SP8 (3X) STED system equipped with a white light source for excitation (tunable excitation from 470–670 nm) and three STED lasers for depletion at 592 nm, 660 nm and 775 nm. Imaging of nuclear staining with DAPI was A 100X/1.4 NA oil immersion objective lens (HCX PL APO STED white, Leica Microsystems) was used for the imaging. Fluorescence signals were passed through a 0.9–1.0 Airy unit pinhole, a tunable AOBS spectral excitation/filtering unit and separate notch (blocking) filters placed in front of integrated hybrid detectors (APD/PMT modules from Hamamatsu Photonics). Detector gain was set to 100 to 200% and gating times from 0.5 to 1.2 ns per images channel. Image frames (1024 × 1024) were acquired sequentially frame-by-frame at a scan speed of 600 lines per second with a pixel size of 28 nm. Raw STED images were deconvoluted with the Huygens software (SVI Netherlands).

### RNA extraction and experimental design

C17.2 cells were differentiated for 1 and 7 days under continuous exposure to CeO_2_ (25 μg/mL), Sm-CeO_2_ (25 μg/mL) or NAC (1 mM). Untreated, proliferating cells (day 0), differentiated cells at day 1 and at day 7 were used as controls. Three replicates were considered for each sample. For RNA extraction, cells were washed after exposure and total RNA was extracted using the RNeasy Mini Columns (Qiagen) according to the manufacturer’s instructions, including a purification step with DNase I treatment. Total RNA concentration was determined spectrophotometrically using NanoDrop (NanoDrop Technologies).

### RNA sequencing and data analysis

The quality control of the mRNA samples was conducted using the Bioanalyzer 2100 (Agilent Technologies) and all samples had RIN values above 9. RNA sequence libraries were generated with standard mRNA stranded protocols from Illumina and sequenced on a Hiseq. 2500 (pair end reads 101 bp long) at the Science for Life Laboratory, Stockholm, Sweden. Data processing was carried out at SNIC-UPPMAX, Uppsala, Sweden^76^. The generated reads were mapped to the mouse genome version GRCm38 using Tophat v. 2.0.4^77^. Read data were converted to gene counts with the program htseq v. 0.5.1^78^ using the ensembl annotation v. 73. Differential gene expression were assessed using linear modeling in R using the bioconductor package Limma that allows for identification of differentially expressed genes in a multifactorial experiment^79^. Genes with an average expression lower than 1 read per sample were removed before analysis of differentially expressed genes. Of the 380561 annotated loci 17794 was expressed above our threshold and retained for analysis of differential gene expression. Only genes with p-values lower than 0.05 after correction for multiple testing (false discovery rate, FDR) and fold change larger than 0.75 on log_2_ scale were considered as differentially expressed. The sequencing data were deposited at ArrayExpress (accession number E-MTAB-4398). Volcano plots were derived by plotting −log_10_(FDR adjusted p-value) in relation to the log_2_(fold change) in R using the *ggplot2* package. Venn diagrams of the differentially expressed genes for each treatment *versus* the untreated control at the same point were plotted with a web-based tool developed by the Bioinformatics & Evolutionary Genomics Laboratory at VIB/UGent, Belgium (http:bioinformatics.psb.ugent.be/webtools/Venn/). The cutoff for the FDR adjusted p-value was set at <0.05 and the cut-off for the log_2_ (fold change) was set at >0.75. The lists of genes for plotting the Venn diagrams were based on the Ensembl gene ID. As part of the quality control a principal component analysis plot was created using rlog-transformed count data; the plot indicated that samples clustered predominantly according to time-point (Supplementary Figure 11).

### Pathway, network and gene enrichment analysis

Ingenuity Pathway Analysis (IPA) (content version 24718999) software (license obtained from Ingenuity Systems, Redwood City, CA) was used to perform canonical pathway analysis, as well as diseases and functions analysis. Heat maps were generated using data output from IPA network and pathway analysis with genes ordered according to average fold change in the control differentiating cells over time. The color coding displayed in the heatmaps shows the directionality of the changes in gene expression, in relation to the control. Gene Ontology (GO) enrichment analysis of the differentially expressed genes was performed using the online tool GOEast^80^ using a Fischer exact test and Alexa’s improved weighted scoring algorithm. The GO enrichment was performed for all the differentially expressed genes together and separately for the up- and downregulated genes. The significance level of enrichment was set to a p-value of 0.05 and the minimum number of genes in the category (q-value) was set at 3 for the up-regulated genes and at 5 for the down-regulated genes. Additionally, GO enrichment was performed for the genes overlapping between CeO_2_ and Sm-doped CeO_2_ as well as CeO_2_ and NAC at both day 1 and day 7.

### Statistical analysis

Differences between groups were evaluated by ANOVA followed by Dunnet’s *post hoc* test (for comparisons *versus* control) or Tukey’s *post hoc* test (for comparisons within groups). For the statistical analysis of the neural growth cone results, the differences between groups were evaluated using the Kruskal-Wallis test (non-parametric) followed by Dunn’s multiple comparison test, as the data were not normally distributed. All analyses were performed using GraphPad Prism version 5.02. P-values < 0.05 were considered as statistically significant.

## Electronic supplementary material

Supporting Figures & Tables
