# Supplementary material for: Cerium oxide nanoparticles inhibit differentiation of neural stem cells
Source: Sci Rep. 2017 Aug 24;7:9284. doi: 10.1038/s41598-017-09430-8 (PMC5570910; doi:10.1038/s41598-017-09430-8)
Supplement: Supplementary file 1 — Supporting Figures & Tables [file 41598_2017_9430_MOESM1_ESM.pdf]

Supporting Information

Cerium oxide nanoparticles inhibit differentiation of neural stem cells

Anda R. Gliga[1,2], Karin Edoff[3], Fanny Caputo[4,5], Thomas Källman[6,7], Hans Blom[8], Hanna L. Karlsson[2], Lina Ghibelli[4], Enrico Traversa[5,9], Sandra Ceccatelli[3], and Bengt Fadeel[1,*]

[1]Division of Molecular Toxicology, and [2]Division of Biochemical Toxicology, Institute of Environmental Medicine, Karolinska Institutet, Stockholm, Sweden; [3]Department of Neuroscience, Karolinska Institutet, Stockholm, Sweden; [4]Department of Biology, and [5]Department of Chemical Science and Technology, University of Rome 'Tor Vergata', Rome, Italy; [6]Department of Medical Biochemistry and Microbiology, Uppsala University, Uppsala, Sweden; [7]Bioinformatics Infrastructure for Life Sciences, Uppsala University, Uppsala, Sweden; [8]Science for Life Laboratory, Royal Institute of Technology, Solna, Sweden; [9]International Research Center for Renewable Energy, Xi'an Jiaotong University, Xi'an China.

[*]To whom correspondence should be addressed. Bengt Fadeel, Division of Molecular Toxicology, Institute of Environmental Medicine, Nobels väg 13, Karolinska Institutet, 171 77 Stockholm, Sweden; Phone: +46 8 524 877 37; Fax: +46 8 34 38 49; E-mail: bengt.fadeel@ki.se

	Dispersion medium	Concentration (µg/mL)	Diameter (nm)	Polydispersity index	Zeta potential (mV)
CeO_2	H_2O	50	nd	nd	-27.5 ± 0.6
		100	264 ± 29	0.333 ± 0.065	nd
	C17.2 medium	20	213 ± 36	0.25 ± 0.04	nd
		50	253 ± 10	0.21 ± 0.01	-9.44 ± 1.38
		100	258 ± 25	0.29 ± 0.02	nd
	Differentiation medium	20	475 ± 0.41	0.410 ± 0.01	nd
		50	553 ± 30	0.403 ± 0.042	-9.72 ± 0.79
		100	795 ± 0.05	0.576 ± 0.05	nd

TEM

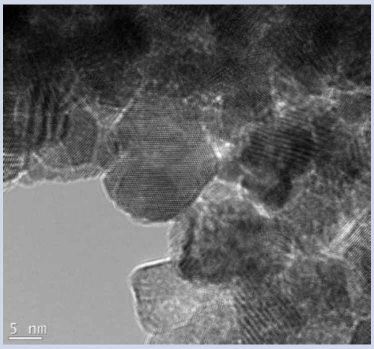

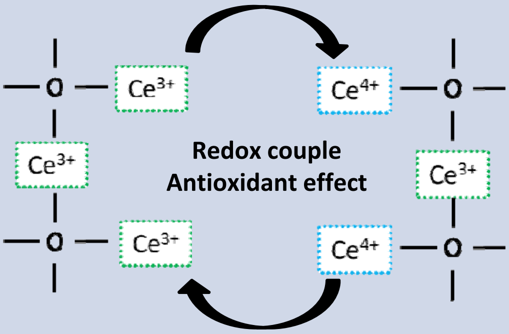

	Dispersion medium	Concentration (µg/mL)	Diameter (nm)	Polydispersity index	Zeta potential (mV)
Sm-CeO_2	H_2O	50	nd	nd	-25.76 ± 0.46
		100	255 ± 6	0.311 ± 0.02	nd
	C17.2 medium	20	131 ± 21	0.248 ± 0.02	nd
		50	167 ± 11	0.34 ± 0.1	-8.81 ± 0.62
		100	177 ± 13	0.374 ± 0.1	nd
	Differentiation medium	20	435 ± 21	0.38 ± 0.01	nd
		50	454 ± 7	0.34 ± 0.01	-9.64 ± 0.29
		100	526 ± 19	0.25 ± 0.02	nd

TEM

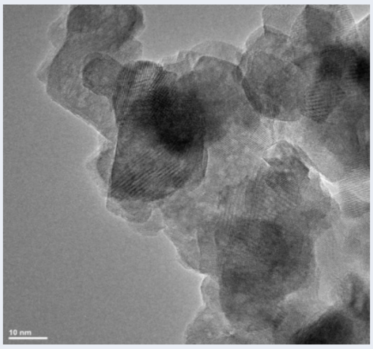

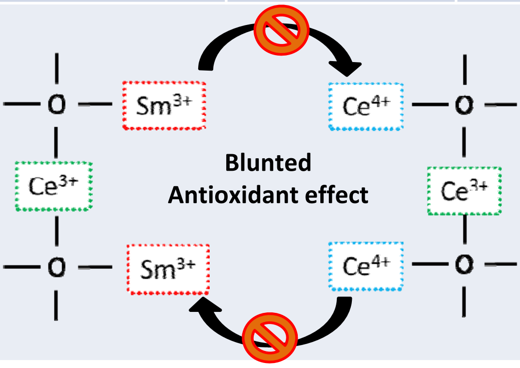

Supplementary figure 1. Physicochemical characterization of CeO_2 and Sm-doped CeO_2 nanoparticles.

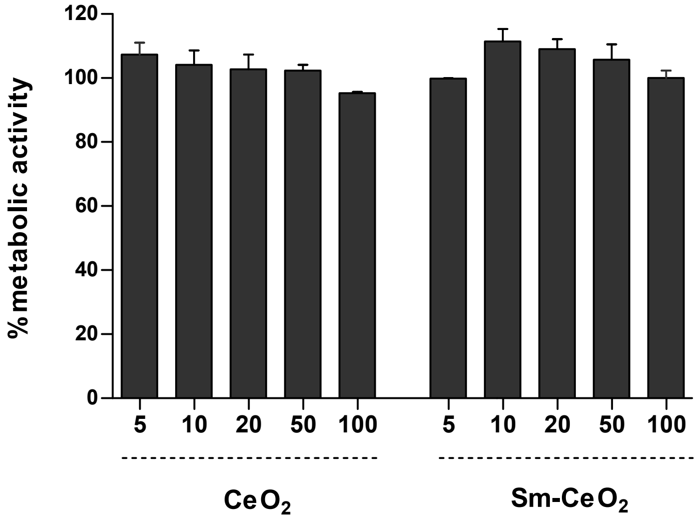

Supplementary figure 2. Cell viability of C17.2 neural progenitor cells after exposure to CeO_2 and Sm-CeO_2 nanoparticles. C17.2 cells were exposed to CeO_2 and Sm-CeO_2 nanoaparticles (5, 10, 20, 50, 100 µg/mL) for 48 h and cellular viability was assessed using the Alamar Blue assay. Results are presented as mean values ± S.D. (n=2).

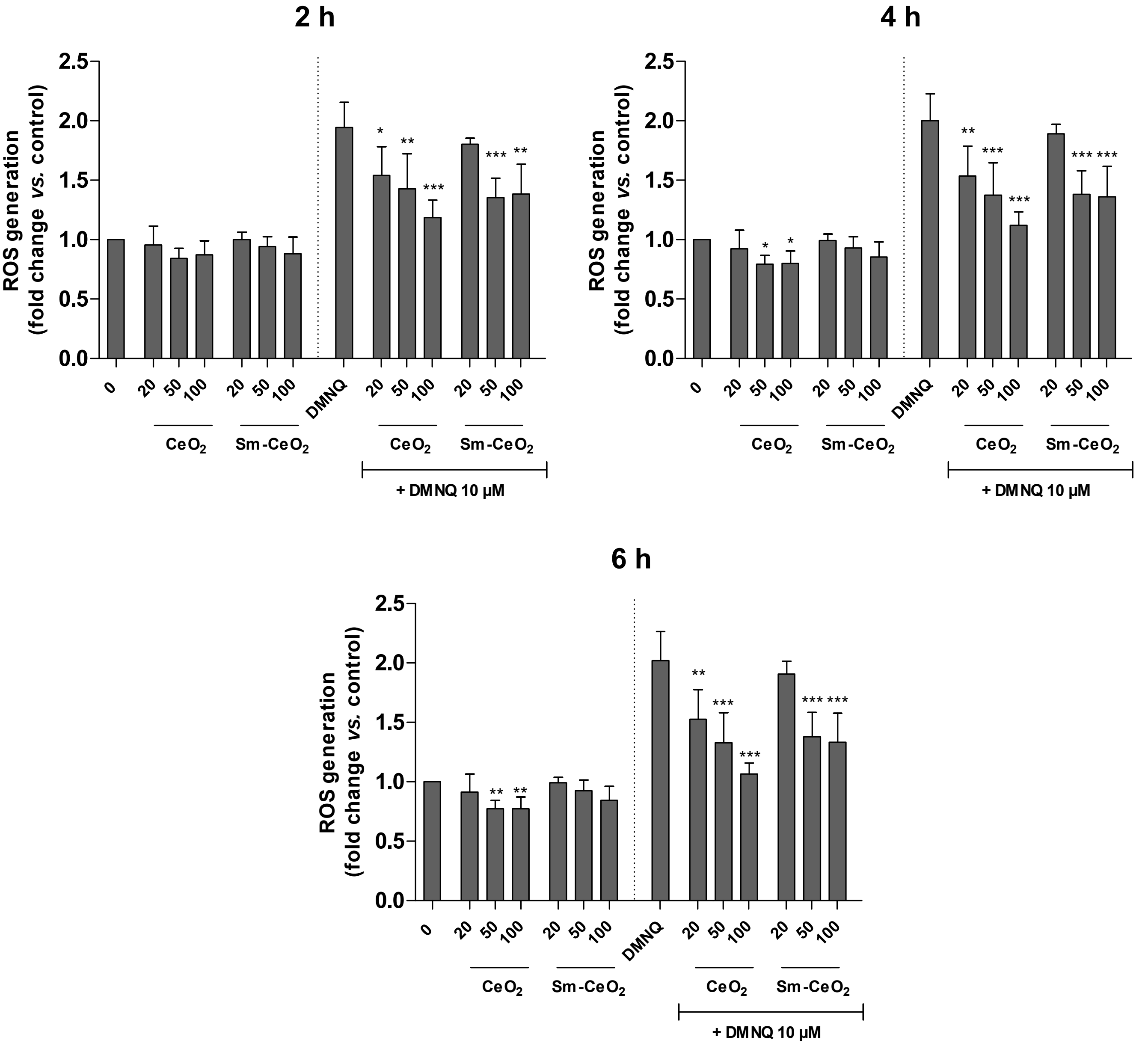

Supplementary figure 3. ROS generation after exposure to the oxidative stress inducer, DMNQ in the presence of CeO_2 or Sm-CeO_2 nanoparticles was investigated using the DCFH-DA assay. Cells were incubated with CeO_2 or Sm-CeO_2 (20, 50, 100 µg/mL) for 4 h, loaded with DCFH-DA and the challenged with DMNQ (10 µM). ROS formation was assessed on a plate reader (Ex 485 nm/Em 535 nm) after 2, 4, and 6 h. ROS induction was expressed as fold change *versus* the control. Results are presented as mean values ± S.D. (n=5). Significant results are indicated with asterisks (* p-value <0.05, ** p-value <0.01, *** p-value <0.001).

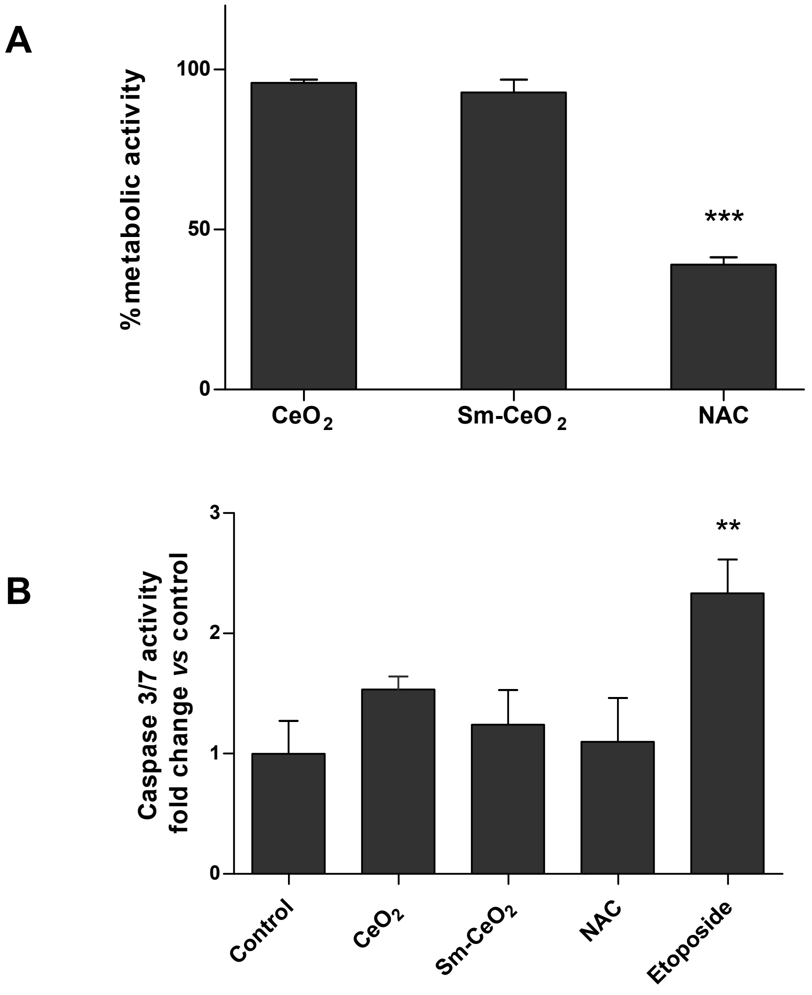

Supplementary figure 4. Cell viability/proliferation of differentiating C17.2 cells. (A) C17.2 cells were differentiated for 7 days in the presence of CeO_2 NPs (25 µg/mL), Sm-CeO_2 NPs (25 µg/mL) and NAC (1 mM). Cell viability/proliferation was assessed using Alamar Blue assay. Results are presented as mean ± S.D.(n=2). Significant results are marked with asterisks (*** for p-value <0.001). (B) Assessment of caspase activation in C17.2 cells differentiated for 7 days in the presence of CeO_2 (25 µg/mL), Sm-CeO_2 (25 µg/mL) or NAC (1 mM). Etoposide (100 µM) was used as a positive control. After exposure, cells lysates were incubated with the fluorogenic caspase substrate, DEVD-AMC and the enzyme catalyzed release of AMC was quantified as described in Methods. Results were normalized according to protein content and are presented as mean values ± S.D. (n=3 for control, CeO_2, Sm-CeO_2, NAC; n=2 for etoposide). Significant results are marked with asterisks (** for p-value <0.01).

Supplementary figure 5.

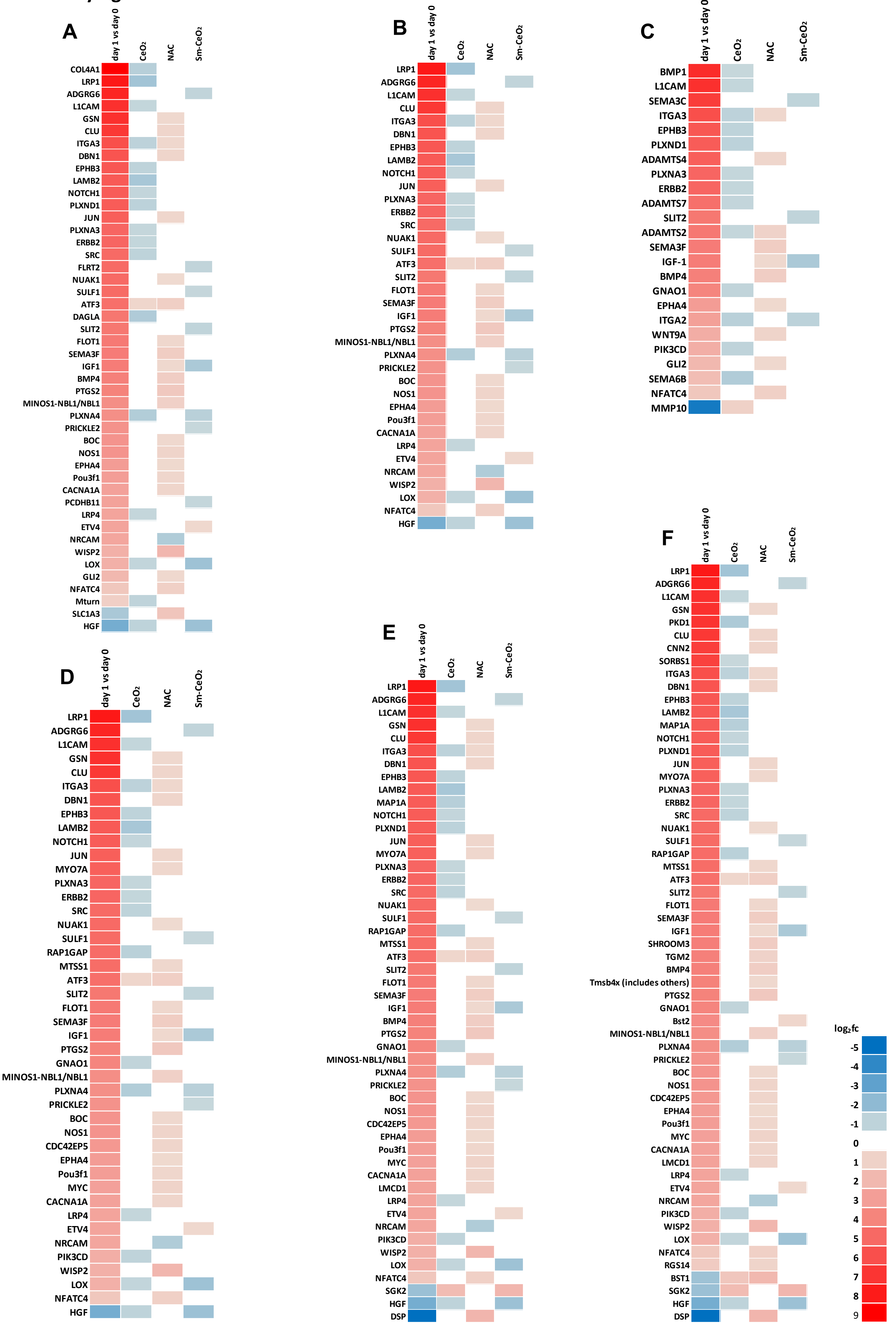

Supplementary figure 6.

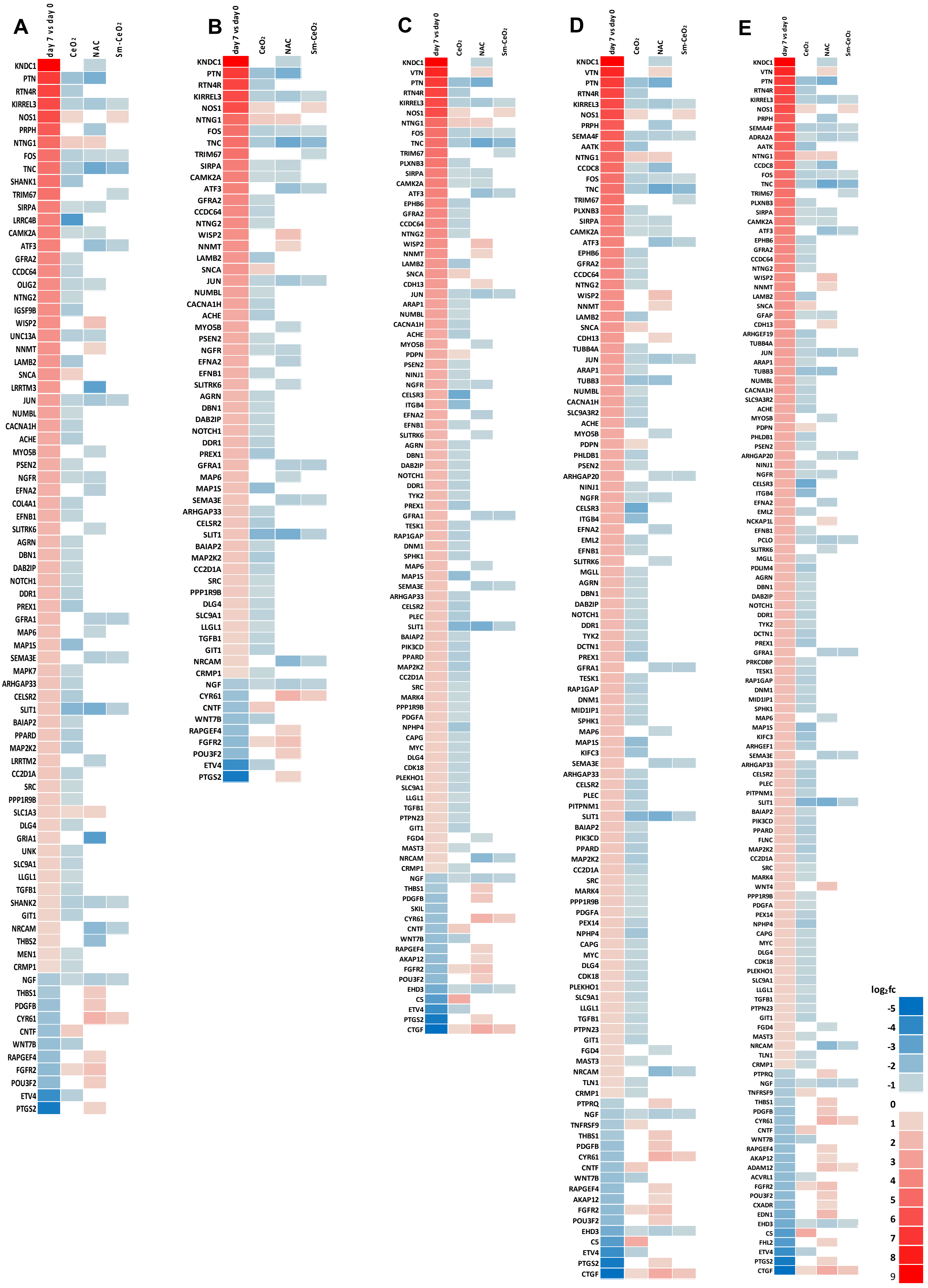

Supplementary figure 5. Heatmaps of selected differentially enriched pathways and networks at differentiation day 1. Development of neurons network (A), Neuritogenesis network (B), Axonal guidance signalling pathway (C), Formation of cellular protrusions network (D), Microtubule dynamics network (E), Organisation of the cytoskeleton network (F) are illustrated. The color coding shows the directionality of the changes in gene expression for cells treated with CeO_2 or Sm-CeO_2 nanoparticles, or NAC, in relation to the control (i.e., differentiating cells). The gene lists have been filtered so that they contain genes that were significantly altered in the control over time (day 1 *versus* day 0, log_2 (fold change) > 0.75) and in at least one of the treatments.

Supplementary figure 6. Heatmaps of selected differentially enriched pathways and networks at differentiation day 7. Development of neurons network (A), Neuritogenesis network (B), Formation of cellular protrusions network (C), Microtubule dynamics network (D), Organisation of the cytoskeleton network (E) are illustrated. The color coding shows the directionality of the changes in gene expression for cells treated with CeO_2 or Sm-CeO_2 nanoparticles, or NAC, in relation to the control (i.e., differentiating cells). The gene lists have been filtered so that they contain genes that were significantly altered in the control over time (day 7 *versus* day 0, log_2 (fold change) > 0.75) and in at least one of the treatments.

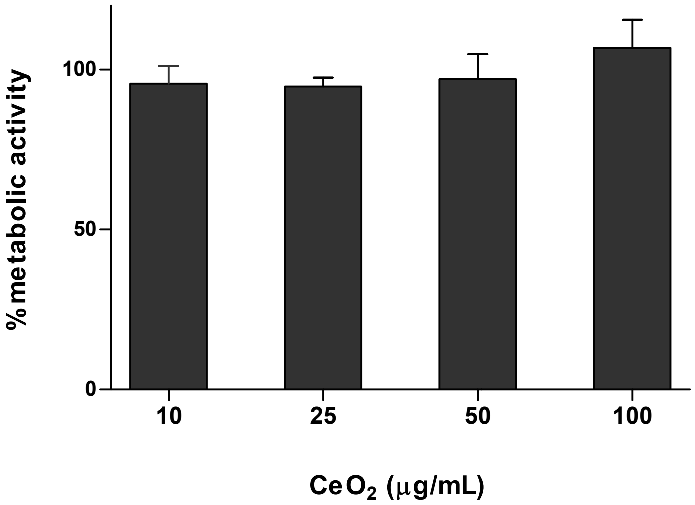

Supplementary figure 7. Cell viability of differentiated C17.2 cells exposed to CeO_2. C17.2 cells were differentiated for 6 days and then exposed to CeO_2 NPs (10 – 100 µg/mL) for 24 h. Cell viability was assessed using Alamar Blue assay. Results are presented as mean values ± S.D.(n=4).

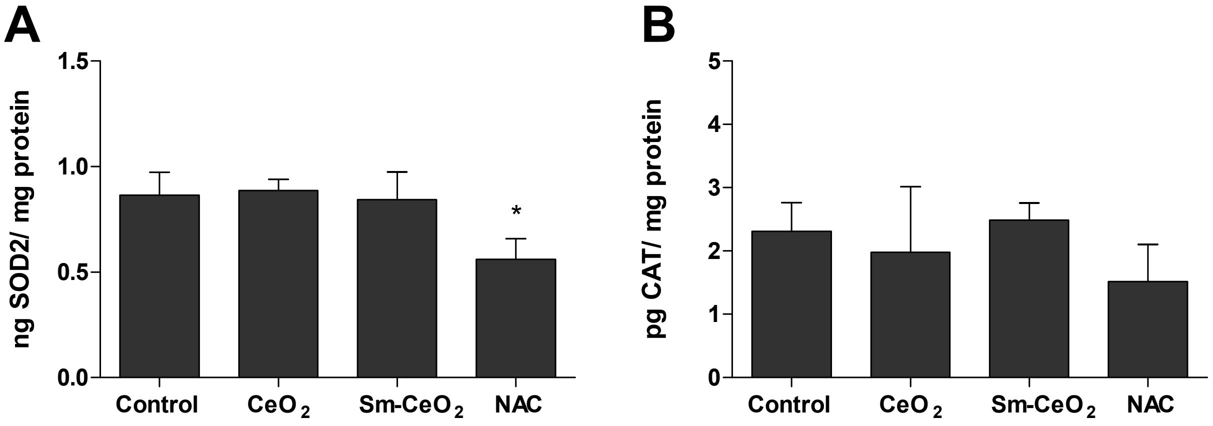

Supplementary figure 8. Expression of antioxidant enzymes superoxide dismutase - SOD2 (A) and catalase – CAT (B) was quantified by ELISA. C17.2 cells were treated with CeO_2 (25 µg/mL), Sm-CeO_2 (25 µg/mL) or NAC (1 mM) and differentiated for 7 days. ELISA was performed on cell lysates and results were normalized according to protein content. Results are presented as mean values ± S.D. (n=3). Significant results are indicated with asterisks (* p-value <0.05)

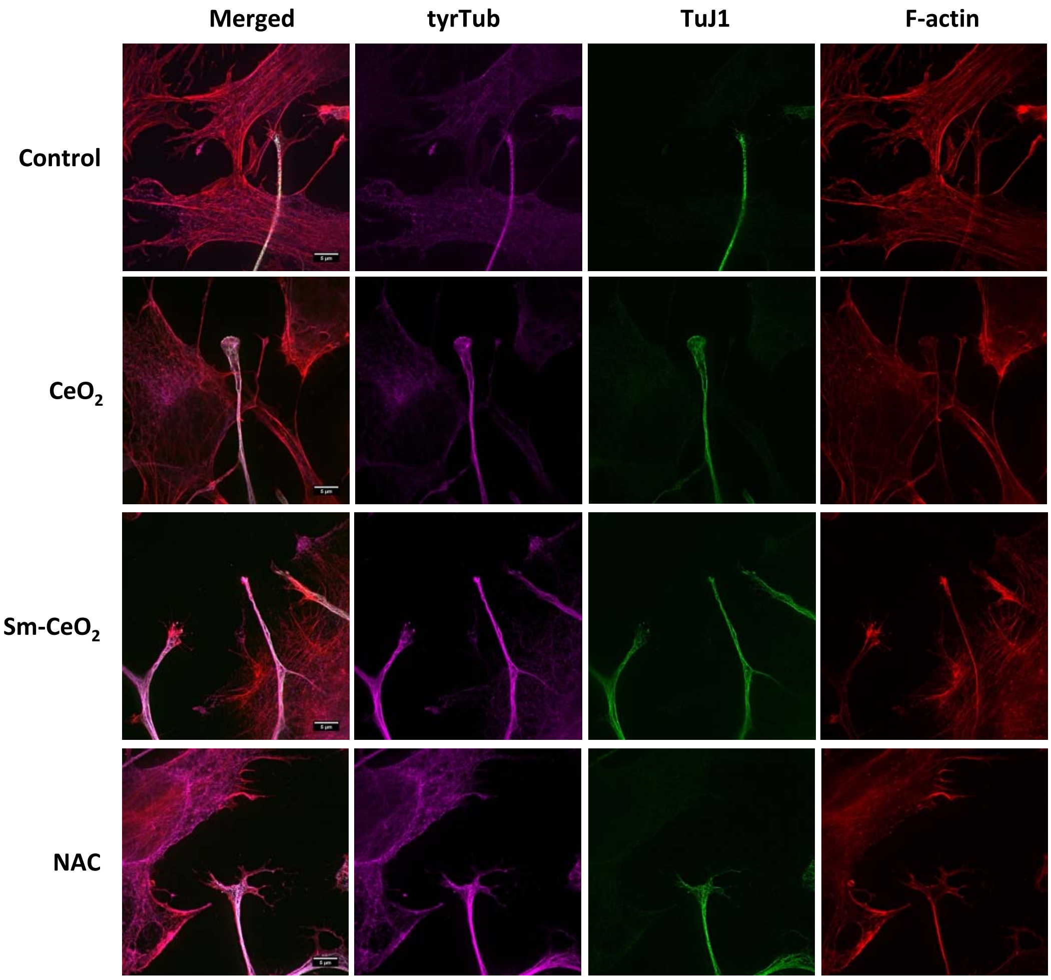

Supplementary figure 9. Structured illumination microscopy (SIM) imaging of neuronal growth cones. By SIM microscopy we investigated the structure of the growth cones. C17.2 cells were differentiated for 6 days in the presence of CeO_2 (25 μg/mL), Sm-CeO_2 (25 μg/mL) or NAC (1 mM). After exposure, cells were fixed and stained for β3-tubulin (anti-TuJ1 antibody, green), tyrosinated tubulin (ABT171 antibody, magenta) and F-actin (phalloidin-TRITC, red).

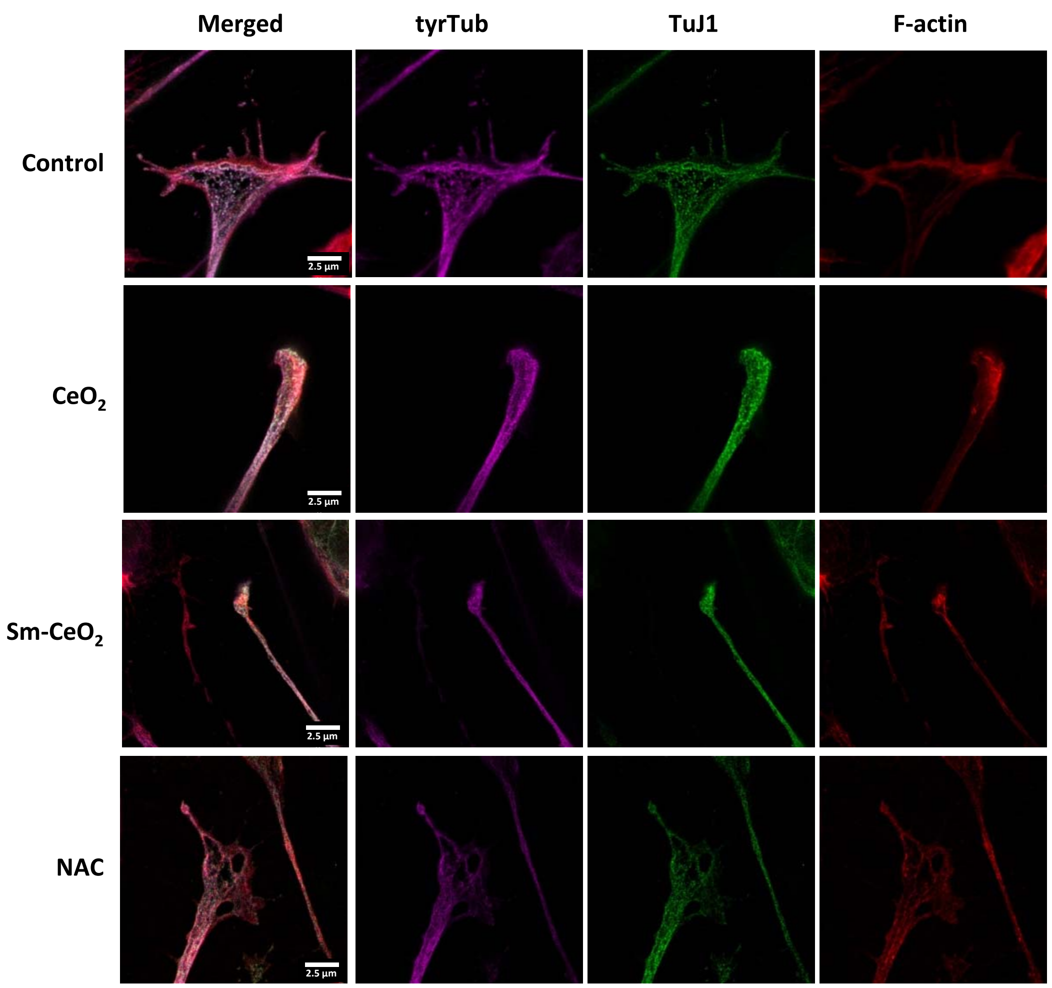

Supplementary figure 10. Stimulated emission depletion (STED) microscopy imaging of neuronal growth cones. By STED microscopy we investigated the structure of the growth cones. C17.2 cells were differentiated for 6 days in the presence of CeO_2 (25 µg/mL), Sm-CeO_2 (25 µg/mL) or NAC (1 mM). After exposure, cells were fixed and stained for β3-tubulin (anti-TuJ1 antibody, green), tyrosinated tubulin (ABT171 antibody, magenta) and F-actin (phalloidin-TRITC, red).

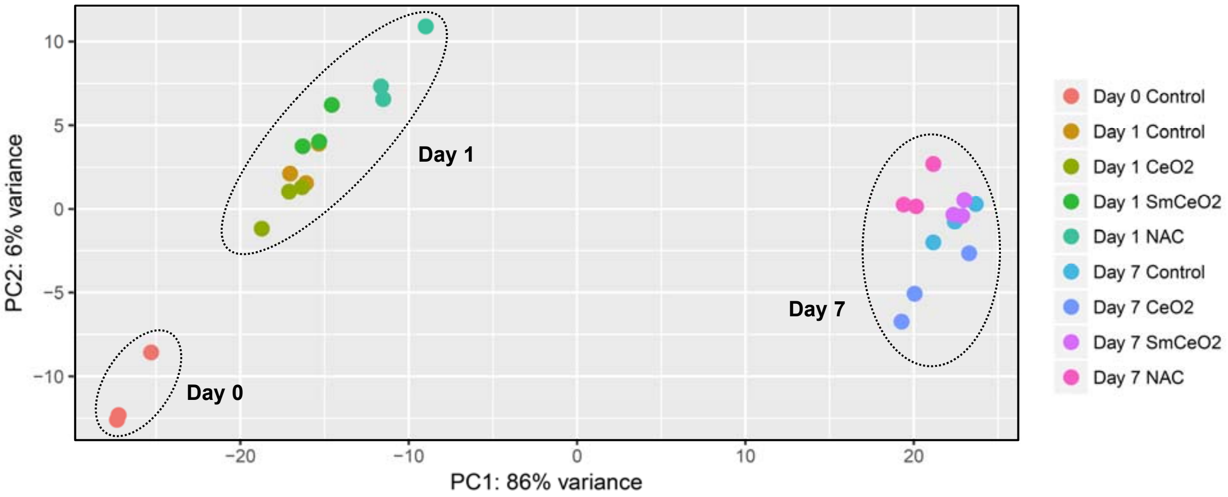

Supplementary figure 11. Principal component analysis was performed on the rlog transformed count data from the RNA-Seq. Results show that samples cluster mainly according to the differentiation time-point.

Supplementary table 1. Top 20 IPA canonical pathways of CeO_2 *vs* Control at Day 1 and Day 7.

Top 20 IPA Pathways CeO_2 vs Control		
**Day 1**		
**Ingenuity Canonical Pathways**	**-log(p-value)**	**z-score**
Caveolar-mediated Endocytosis Signaling	5,79	NaN
Axonal Guidance Signaling	5,63	NaN
Molecular Mechanisms of Cancer	5,61	NaN
Virus Entry via Endocytic Pathways	5,60	NaN
Agrin Interactions at Neuromuscular Junction	5,05	-2,121
Hepatic Fibrosis / Hepatic Stellate Cell Activation	4,74	NaN
Epithelial Adherens Junction Signaling	4,67	NaN
Reelin Signaling in Neurons	4,52	NaN
ILK Signaling	4,08	-1,291
Signaling by Rho Family GTPases	3,98	-3
Ephrin Receptor Signaling	3,83	NaN
Estrogen-Dependent Breast Cancer Signaling	3,73	-2,121
Paxillin Signaling	3,61	-2,828
EIF2 Signaling	3,56	1,414
Remodeling of Epithelial Adherens Junctions	3,50	NaN
PI3K Signaling in B Lymphocytes	3,37	-0,302
RhoGDI Signaling	3,31	2,887
NF-κB Activation by Viruses	3,29	NaN
Serotonin Receptor Signaling	3,09	NaN
Germ Cell-Sertoli Cell Junction Signaling	3,09	NaN
**Day 7**		
**Ingenuity Canonical Pathways**	**-log(p-value)**	**z-score**
Hepatic Fibrosis / Hepatic Stellate Cell Activation	7,20	NaN
Axonal Guidance Signaling	7,05	NaN
14-3-3-mediated Signaling	6,60	-2,309
Role of Macrophages, Fibroblasts and Endothelial Cells in Rheumatoid Arthritis	4,88	NaN
Epithelial Adherens Junction Signaling	4,60	NaN
Sertoli Cell-Sertoli Cell Junction Signaling	4,53	NaN
iNOS Signaling	3,77	-2,646
GNRH Signaling	3,57	-2,138
STAT3 Pathway	3,51	-1,897
RANK Signaling in Osteoclasts	3,44	-2,53
PDGF Signaling	3,32	-3,162
CD27 Signaling in Lymphocytes	3,25	-1,89
Aryl Hydrocarbon Receptor Signaling	3,20	-1
PPAR Signaling	3,20	3,317
Erythropoietin Signaling	3,15	NaN
4-1BB Signaling in T Lymphocytes	3,10	-0,816
Remodeling of Epithelial Adherens Junctions	3,10	-2
PI3K Signaling in B Lymphocytes	3,08	-3,051
Wnt/Ca^{2+} pathway	3,03	-2,121
Xenobiotic Metabolism Signaling	2,98	NaN

Pathway analysis was performed in IPA on the differentially expressed genes of the contrasts CeO_2 *versus* Control at day 1 and at day 7. Top20 significantly enriched canonical pathways at day 1 and at day 7 are illustrated, ordered according to the statistical significance (-log(p-value)). Some pathways are additionally characterized by z-score, a measure of the activation state of the pathway. NaN, activity pattern not available.

Supplementary table 2. Top 20 IPA canonical pathways of CeO_2 *vs* NAC at Day 1 and Day 7.

Top 20 IPA Pathways CeO_2 vs NAC

Day 1

Ingenuity Canonical Pathways	-log(p-value)	z-score
Axonal Guidance Signaling	9,23	NaN
Hepatic Fibrosis / Hepatic Stellate Cell Activation	9,12	NaN
Epithelial Adherens Junction Signaling	7,77	NaN
Wnt/β-catenin Signaling	5,42	-2,263
Caveolar-mediated Endocytosis Signaling	5,22	NaN
Regulation of the Epithelial-Mesenchymal Transition Pathway	4,81	NaN
Virus Entry via Endocytic Pathways	4,41	NaN
Ephrin Receptor Signaling	4,35	NaN
Molecular Mechanisms of Cancer	4,19	NaN
Sertoli Cell-Sertoli Cell Junction Signaling	4,12	NaN
Chronic Myeloid Leukemia Signaling	4,07	NaN
Signaling by Rho Family GTPases	4,06	-6,252
Adipogenesis pathway	4,02	NaN
ILK Signaling	3,68	-4,333
TGF-β Signaling	3,68	-2,183
RhoA Signaling	3,59	-3,922
Role of Osteoblasts, Osteoclasts and Chondrocytes in Rheumatoid Arthritis	3,59	NaN
Pancreatic Adenocarcinoma Signaling	3,51	-3,710
Mouse Embryonic Stem Cell Pluripotency	3,49	-3,674
Actin Cytoskeleton Signaling	3,39	-5,284

Day 7

Ingenuity Canonical Pathways	-log(p-value)	z-score
Hepatic Fibrosis / Hepatic Stellate Cell Activation	6,58	NaN
Calcium Signaling	3,86	0,000
Leukocyte Extravasation Signaling	3,33	-0,832
Agranulocyte Adhesion and Diapedesis	3,08	NaN
Inhibition of Matrix Metalloproteases	2,93	NaN
Actin Cytoskeleton Signaling	2,91	-2,496
Axonal Guidance Signaling	2,33	NaN
PI3K Signaling in B Lymphocytes	2,19	0,000
Integrin Signaling	2,00	-3,162
Leukotriene Biosynthesis	1,99	NaN
Reelin Signaling in Neurons	1,95	NaN
Synaptic Long Term Potentiation	1,93	-1,000
Paxillin Signaling	1,88	-2,236
ERK/MAPK Signaling	1,86	-1,732
Mitochondrial L-carnitine Shuttle Pathway	1,84	NaN
Signaling by Rho Family GTPases	1,83	-3,207
RhoGDI Signaling	1,72	2,714
cAMP-mediated signaling	1,71	0,577
Granulocyte Adhesion and Diapedesis	1,65	NaN
Caveolar-mediated Endocytosis Signaling	1,65	NaN

Pathway analysis was performed in IPA on the differentially expressed genes of the contrasts CeO_2 *versus* NAC at day 1 and at day 7. Top20 significantly enriched canonical pathways at day 1 and at day 7 are illustrated, ordered according to the statistical significance (-log(p-value)). Some pathways are additionally characterized by z-score, a measure of the activation state of the pathway. NaN, activity pattern not available.

Supplementary table 3. Gene ontology enrichment of the differentially expressed genes of CeO_2 *versus* control which were upregulated at day 7.

	Level	Gene symbols	-log10(p-value)
**BIOLOGICAL PROCESS**			
**amino acid transmembrane transport**	10	Slc7a2 Slc1a3 Slc7a13	2,92
**positive regulation of cysteine-type endopeptidase activity involved in apoptotic process**	10	Ctgf Map3k5 Snca	1,96
**dicarboxylic acid transport**	9	Pdpn Slc1a3 Slc1a7	1,84
**cellular calcium ion homeostasis**	9	Cacnb4 Csrp3 Hc	1,59
**calcium ion transport**	9	Ctgf Slc24a1 Cacnb4	1,30
**CELLULAR COMPONENT**			
**clathrin-coated vesicle**	10	Slc40a1 Rab27b Snca	1,30
**multivesicular body**	9	Slc40a1 Acpp Rab27b	3,55
**cytosol**	7	Rps13 Pstpip2 Ctgf Nos1 Fgf1 Slc12a3 Snca Adh1 Cntf Ppargc1a	1,39
**plasma membrane part**	5	Atp1b1 Pdpn Slc1a3 Fcgr4 Tnfrsf9 Fgfr2 Sytl2 Megf10 Tlr1 Rab27b Slc12a3 Pgm5 Gria4 Il17rb Igsf10 Cldn1 Ntng1 Slc24a1 Enpp2 Slc15a2 Cacnb4 Hc	2,79
**tight junction**	4	Cldn15 Cgn Cldn1 Cldn20	2,81
**MOLECULAR FUNCTION**			
**sodium ion transmembrane transporter activity**	8	Atp1b1 Slc1a3 Slc12a3 Slc24a1 Slc1a7	2,05
**amino acid transmembrane transporter activity**	8	Pdpn Slc7a2 Slc1a3 Slc7a13	1,59
**magnesium ion binding**	5	Map3k5 Pgm5 Snca	1,35
**calcium ion binding**	5	Plcd4 Vsnl1 Snca Enpp2 Svep1 Galnt3	1,33
**growth factor activity**	4	Ctgf Ogn Fgf1 Cntf Il5	3,21

Gene ontology enrichment was performed as described in Methods. Top5 ontologies for each domain are illustrated, ordered according to the hierarchical level, together with the corresponding genes. The cutoff for $-\log_{10}$(p-value) was set at > 1.3, and the cutoff for the number of genes in a category was set at >2.

Supplementary table 4. Gene ontology enrichment of the differentially expressed genes of CeO_2 *versus* control which were downregulated at day 7.

	Level	Gene symbols	-log10(p-value)
**BIOLOGICAL PROCESS**			
**positive regulation of Ras GTPase activity**	13	Scrib Arhgef19 Arhgap27 Jun Agrn Dab2ip Arap1 Llgl1	2,49
**neuron projection regeneration**	12	Rtn4rl2 Lamb2 Tnc Jun Gfap	2,34
**negative regulation of protein tyrosine kinase activity**	11	Mvp Hyal2 Psen2 Srcin1 Zfyve28	4,35
**regulation of dendrite development**	11	Shank1 Prex1 Ache Dab2ip Camk2b Srcin1 Numbl	2,13
**negative regulation of protein serine/threonine kinase activity**	11	Hyal2 Pkig Lrp5 Dab2ip Hexim1 Men1	1,55
**axon ensheathment**	11	Mir23b Ppard Gal3st1 Olig2 Tgfb1 Myrf Ercc2	1,45
**neuron projection morphogenesis**	11	Celsr3 Slit1 Rtn4rl2 Map1s Tubb3 Shank1 Lamb2 Tnc Rtn4r Celsr2 Sema4f Wnt7b Etv4 Jun Artn Notch1 Efnb1 Dlg4 Apbb1 Dab2ip Ephb2 Ank3	1,43
**angiogenesis**	10	Foxs1 Col18a1 Hspg2 Plxnd1 Vash1 Col4a2 Col4a1 Arhgap22 Jun Plcd3 Nr4a1 Notch1 Mmp19 Dab2ip Eng Ephb2 Adam15 Myh9 Ecm1 Shb	5,96
**positive regulation of gliogenesis**	10	Tspo Sox10 Clcf1 Olig2 Notch1 Rela Gfap	3,71
**negative regulation of cysteine-type endopeptidase activity involved in apoptotic process**	10	Sfn Ints1 Nr4a1 Por Src Rps6ka1	2,03
**CELLULAR COMPONENT**			
**transcription factor complex**	12	Foxs1 Npas4 Zfpm1 Tceb2 Edf1 Tcf3 Taf1c Nfatc2 Fos Tada3 Foxp4 Jun	3,10
**nucleoplasm part**	11	Hspa1a Foxs1 Npas4 Zfpm1 Ncor2 Tceb2 Ints1 Hr Edf1 Tcf3 Taf1c Nfatc2 Fos Tada3 Foxp4 Hdac5 Isg20 Cxxc1 Jun Olig2 Pagr1a Nr4a1 Myc Mapk7 Npas2 Ercc2 Rela Zc3h3 Tfip11 Polr2l Ell Arid5a Spen	1,86
**intrinsic to endoplasmic reticulum membrane**	10	Abcb9 Sppl2b Extl1 Emc10 Tap2 Lrmp Dgat2 Srebf2	1,67
**cortical cytoskeleton**	9	Nos2 Trpv4 Dlg4 Slc2a1 Tmod1 Myh9 Llgl1 Ppp1r9b	3,57
**endocytic vesicle**	9	Anxa11 Hyal2 Dab2ip Myh9 Ngfr Ehd3	1,53
**actin cytoskeleton**	8	Espn Aire Myh14 Hspb7 Trpv4 Ablim2 Nfatc2 Ankrd23 Flnc Triobp Zyx Whrn Myl6b Vps18 Myo18b Myo18a Gas2l1 Baiap2 Slc2a1 Tln1 Dbn1 Arpc1b Myh9 Fhod1 Aldoa Dctn3 Baiap2l1 Cdc42bpb Srcin1 Llgl1 Pdlim7	5,83
**cytoskeletal part**	8	Ccdc85b Klc3 Map1s Tubb3 Tppp3 Lzts2 Kifc3 Espn Nphp4 Shank1 Cdh23 Map3k11 Myh14 Anxa11 Lmna Map2k2 Fsd1 Dctn1 Cep170b Trpv4 Shank2 Snph Tada3 Eml2 Zyx Whrn Mid1ip1 Myl6b Clip2 Ccdc64 Grik5 Axin2 Vps18 Myo18b Dnm1 Lrrc45 Myo18a Tubb4a Gas2l1 Dlg4 Slc2a1 Myc Map1lc3a Sema4c Tubb6 Poc1a Tln1 Arpc1b Tubb2a Myh9 Ercc2 Ptpn23 Fhod1 Dctn3 Camk2b Fbxl7 Psen2 Ptch1 [illegible]	4,93
**ionotropic glutamate receptor complex**	8	Shank1 Shank2 Grik5 Dlg4 Cpt1c	2,16
**early endosome**	8	Map2k2 Mib2 Atp9a Furin Vps18 Ptpn23 Hgs Llgl1 Zfyve28	2,03
**microtubule cytoskeleton**	8	Ccdc85b Klc3 Map1s Tubb3 Tppp3 Lzts2 Kifc3 Nphp4 Cdh23 Map3k11 Anxa11 Map2k2 Fsd1 Dctn1 Cep170b Trpv4 Snph Tada3 Eml2 Mid1ip1 Clip2 Ccdc64 Axin2 Dnm1 Lrrc45 Tubb4a Gas2l1 Myc Map1lc3a Tubb6 Poc1a Tln1 Tubb2a Myh9 Ercc2 Ptpn23 Mc1r Dctn3 Camk2b Fbxl7 Mypn Psen2 Cep250 Mark4 Kif17 Rps6ka1	1,76
**ATP binding**	8	Atp1a3 Mapk15 Aatk Kifc3 Abcc6 Abcb9 Jak3 Galk1 Map3k11 Myh14 Pfkl Map2k2 Atp13a2 Csnk1g2 Atp9a Trpv4 Acacb Epha10 Ddx54 Pik3cd Ephb6 Sars2 D8Ertd82e Ddr1 Sphk1 Tesk1 Dyrk1b Tap2 Pex6 Acsbg1 Myo18b Hspa2 Pak4 Map3k10 Cdk18 Dcakd Abcc10 Myo18a Srpk3 Tyk2 Pkn3 Dgkz Ephb2 Mapk7 Pfkfb4 Trpm4 Dmpk Skiv2l Myh9 Ercc2 Ighmbp2 Camk2b Entpd2 Ehd3 Trpv1 Acvrl1 Src Mark4 Cdc42bpb Nod1 Ddx49 Kif17 Rps6ka1 Mast3 Camk2a Stk10 Nav2	4,98
**MOLECULAR FUNCTION**			
**histone-lysine N-methyltransferase activity**	8	Cxxc1 Dot1l Setd1b Kmt2b Men1	2,46
**motor activity**	7	Klc3 Kifc3 Myh14 Dctn1 Myl6b Myo18a Myh9 Kif17	1,58
**zinc ion binding**	6	Zfp385c Car9 Adam11 Aire Esrra Pdlim4 Adamtsl1 Ppard Rai1 Mib2 Zdhhc8 Trim47 Ablim2 Cda Lims2 Zmiz2 Crip2 Git1 Kdm4b Nr1h2 Rara Fasn Zyx Zswim4 Cxxc1 Unk Nr4a1 Zfp598 Mmp17 Mmp19 Cpa4 Zdhhc12 Haghl Zdhhc18 Adam15 Adamts7 Trim46 Ighmbp2 Unkl Polr2l Zfp276 Arap1 Ptch1 Zswim8 Kmt2b Pdlim7 Nr1d1 Man2c1	4,42
**transcription regulatory region DNA binding**	6	Cebpd Fosl1 Irf7 Aire Ncor2 Hlx Sox10 Cdx1 Tcf3 Srebf1 Nfatc2 Fos	3,43
**protein serine/threonine kinase activity**	6	Mapk15 Aatk Map3k11 Map2k2 Csnk1g2 Tesk1 Dyrk1b Pak4 Map3k10 Cdk18 Srpk3 Pkn3 Eng Mapk7 Dmpk Ercc2 Camk2b Trpv1 Adck4 Acvrl1 Mark4 Cdc42bpb Rps6ka1 Mast3 Camk2a Stk10	2,65
**protein tyrosine kinase activity**	6	Aatk Jak3 Map3k11 Map2k2 Epha10 Ephb6 D8Ertd82e Ddr1 Tesk1 Dyrk1b	2,23
**sequence-specific DNA binding**	5	Foxs1 Cebpd Fosl1 Irf7 Ncor2 Snai1 Esrra Hlx Sox10 Cdx1 Ppard Edf1 Tcf3 Sox13 Srebf1 Nfatc2 Fos Etv4 Foxp4 Hdac5 Nr1h2 Erf Rara Cc2d1a Cxxc1 Kdm6b Prrx2 Jun Nr4a1 Myrf Jdp2 Notch1 Per1 Myc Mkl1 Rela	4,05
**cytokine receptor activity**	5	Il3ra Cntfr Epor Csf2ra Il12rb1 Il18rap Il17rc Gfra2	2,90
**calcium ion binding**	5	Celsr3 Slit1 Cdh15 Celsr1 Ltbp4 Cdh23 Fbln1 Anxa11 Egfl8 Celsr2 Creld1 Notch3 Pclo Myl6b Padi3 Plcd3 Mmp17 Notch1 Mmp19 Agrn Efhd2 Mgp Cadps Ehd3 Plcd1 Nkd2 Plcb3 Padi2	2,90

Gene ontology enrichment was performed as described in Methods. Top10 ontologies for each domain are illustrated, ordered according to the hierarchical level, together with the corresponding genes. The cutoff for $-\log_{10}$(p-value) was set at > 1.3, and the cutoff for the number of genes in a category was set at >4.

Supplementary table 5. Gene ontology enrichment of the overlapping genes between the two particles (CeO_2 and Sm-CeO_2) at Day 1 and Day 7 ordered by the hierarchical level.

Day 1

GOID	Ontology	Term	Level	p
GO:0016567	biological_process	protein ubiquitination	9	0,0229143
GO:0005635	cellular_component	nuclear envelope	9	0,0428321
GO:0008305	cellular_component	integrin complex	8	0,0001266
GO:0001568	biological_process	blood vessel development	8	0,0260721
GO:0090100	biological_process	positive regulation of transmembrane receptor protein ser	7	0,0018975
GO:0030335	biological_process	positive regulation of cell migration	7	0,0106317
GO:0006468	biological_process	protein phosphorylation	7	0,0110999
GO:0043523	biological_process	regulation of neuron apoptotic process	7	0,0222785
GO:0052548	biological_process	regulation of endopeptidase activity	7	0,0437401
GO:0005794	cellular_component	Golgi apparatus	7	0,0514622
GO:0043066	biological_process	negative regulation of apoptotic process	7	0,0538275
GO:0007229	biological_process	integrin-mediated signaling pathway	6	0,0032296
GO:0004674	molecular_function	protein serine/threonine kinase activity	6	0,0118817
GO:0016323	cellular_component	basolateral plasma membrane	6	0,0195441
GO:0016324	cellular_component	apical plasma membrane	6	0,0437572
GO:0008236	molecular_function	serine-type peptidase activity	5	0,0052437
GO:0032550	molecular_function	purine ribonucleoside binding	5	0,005462
GO:0006508	biological_process	proteolysis	5	0,0071894
GO:0005737	cellular_component	cytoplasm	5	0,0814567
GO:0051101	biological_process	regulation of DNA binding	4	0,0016139
GO:0005178	molecular_function	integrin binding	4	0,0017465
GO:0046982	molecular_function	protein heterodimerization activity	4	0,0129827
GO:0060249	biological_process	anatomical structure homeostasis	4	0,0276623
GO:0030198	biological_process	extracellular matrix organization	4	0,0606192
GO:0045178	cellular_component	basal part of cell	3	0,000309
GO:0048771	biological_process	tissue remodeling	3	0,0010753
GO:0097458	cellular_component	neuron part	3	0,005472
GO:0005578	cellular_component	proteinaceous extracellular matrix	3	0,0057226
GO:0009986	cellular_component	cell surface	3	0,0154978
GO:0007155	biological_process	cell adhesion	3	0,0287634
GO:0045121	cellular_component	membrane raft	3	0,0420264
GO:0043167	molecular_function	ion binding	2	7,48E-08
GO:0044420	cellular_component	extracellular matrix part	2	0,0249753
GO:0043226	cellular_component	organelle	1	0,0344979

Day 7

GOID	Ontology	Term	Level	p
GO:0045893	biological_process	positive regulation of transcription, DNA-dependent	9	0,002306
GO:0001934	biological_process	positive regulation of protein phosphorylation	9	0,074213
GO:0055072	biological_process	iron ion homeostasis	7	0,001409
GO:0043066	biological_process	negative regulation of apoptotic process	7	0,00221
GO:0005829	cellular_component	cytosol	7	0,014598
GO:0016324	cellular_component	apical plasma membrane	6	0,019627
GO:0030334	biological_process	regulation of cell migration	6	0,053359
GO:0006936	biological_process	muscle contraction	5	0,013549
GO:0008284	biological_process	positive regulation of cell proliferation	5	0,021558
GO:0009408	biological_process	response to heat	4	0,001317
GO:0008083	molecular_function	growth factor activity	4	0,009005
GO:0042127	biological_process	regulation of cell proliferation	4	0,070141
GO:0005615	cellular_component	extracellular space	3	0,006688
GO:0005539	molecular_function	glycosaminoglycan binding	3	0,010359
GO:0042802	molecular_function	identical protein binding	3	0,063495
GO:0006955	biological_process	immune response	2	0,040777
GO:0044456	cellular_component	synapse part	2	0,055098
GO:0005576	cellular_component	extracellular region	1	0,035131

Gene ontology enrichment was performed as described in Methods on the genes shared by the two nanoparticles at day 1 (A) and at day 7 (B). Significantly enriched ontologies are illustrated, ordered according to the hierarchical level. The cutoff for p-value was set at < 0.05, and the cutoff for the number of genes in a category was set at >2.
